# Lithocholic acid induces T3SS-dependent formation of invasion-competent *Shigella flexneri* aggregates

**DOI:** 10.1128/iai.00665-25

**Published:** 2026-05-29

**Authors:** Jonah Lanier, Jaden J. Skelly, Freddie Salsbury, Volkan K. Köseoğlu

**Affiliations:** 1 Department of Microbiology and Immunology, Wake Forest University School of Medicine 12279, Winston-Salem, North Carolina, USA; 2 Department of Physics, Wake Forest University 8676, Winston-Salem, North Carolina, USA; University of California San Diego School of Medicine, La Jolla, California, USA

**Keywords:** *Shigella flexneri*, lithocholic acid, type III secretion system, bacterial aggregation, invasion

## Abstract

*Shigella flexneri* causes shigellosis, the second leading cause of diarrheal deaths worldwide. The pathogen invades colonic epithelial cells using a type III secretion system (T3SS) that delivers effector proteins to remodel the host actin cytoskeleton. Following invasion, *S. flexneri* acquires actin-based motility and spreads cell to cell, driving epithelial destruction and bloody diarrhea. These intracellular infection processes have been investigated primarily using exponentially growing planktonic bacteria. However, recent animal studies revealed that *S. flexneri* also forms multicellular aggregates in the colonic lumen, yet the function of this extracellular phase remains unclear. Here, we show that lithocholic acid (LCA), an abundant secondary bile acid in the colon, acts as a potent signal that induces *S. flexneri* aggregation at physiological concentrations (≥50 µM). LCA-induced aggregation depends on the T3SS and its tip protein IpaD, which is required to initiate aggregate formation. LCA-induced aggregates are capable of invasion by eliciting actin remodeling and elevated T3SS activation during early interactions with colonic epithelial HT-29 and Caco-2 cells. These findings identify LCA as a luminal cue that links extracellular aggregation to intracellular infection through a new aggregate-mediated mode of epithelial invasion.

## INTRODUCTION

*Shigella* spp. are the causative agents of bacillary dysentery, or shigellosis, a highly contagious diarrheal disease characterized by abdominal cramps, fever, severe diarrhea, and stools containing mucus and blood (1, 2). Globally, shigellosis accounts for an estimated 270 million cases and 200,000 deaths annually, disproportionately affecting children under 5, with 64,000 deaths and long-term consequences such as persistent diarrhea and growth faltering (3–7). Despite ongoing vaccine development efforts, no licensed vaccine is currently available (8, 9). The World Health Organization recently designated *Shigella* spp. as a “high priority” pathogen due to rising antimicrobial resistance (10).

Among *Shigella* spp., *S. flexneri* is the predominant cause of shigellosis, responsible for 65% of pediatric and 60% of all cases worldwide (6, 11). It is highly infectious, with as few as 100 bacteria sufficient to cause disease (12). Transmission occurs through person-to-person contact, ingestion of contaminated food or water, and, in some cases, sexual contact (1). Following transmission, *S. flexneri* ultimately invades the colonic epithelium, where it causes mucosal ulceration marked by inflammation, vascular damage, and epithelial detachment, hallmarks confirmed in human biopsy studies (13–16). Invasion of epithelial cells relies on the type III secretion system (T3SS) of the pathogen, which delivers effector proteins into host cells to subvert cellular processes (17). Once inside the cytosol, *S. flexneri* uses the polar surface protein IcsA to acquire actin-based motility, enabling intracellular movement (18, 19). Actin-based motility, combined with effector-driven manipulation of the plasma membrane, enables the pathogen to disseminate across the colonic epithelium (20, 21).

While the intracellular phase of *S. flexneri* infection has been extensively studied, mechanisms supporting the extracellular phase remain poorly understood. The low infectious dose and an incubation period of up to 4 days (1, 22) suggest that *S. flexneri* establishes extracellular colonization in the colon. Supporting this, recent studies in infant rabbit and mouse models have shown that *S. flexneri* replicates extracellularly and forms dense aggregates in the colonic lumen (23, 24).

One class of luminal molecules that may influence this early colonization phase is bile acids (25). These detergent-like compounds are synthesized from cholesterol in the liver as primary bile acids, stored in the gallbladder, and secreted into the small intestine within bile following food intake (26). There, they facilitate dietary lipid digestion and absorption (27). Approximately 95% of bile acids undergo enterohepatic circulation, while the 5% remains in the intestines, where gut microbiota convert primary bile acids into secondary bile acids (28). As a result, bile acid composition shifts dramatically between the small intestine and the colon. Primary bile acids, cholic acid and chenodeoxycholic acid, dominate in the small intestine, whereas secondary bile acids, deoxycholic acid (DCA) and lithocholic acid (LCA), are more abundant in the colon (29). Beyond their role in lipid digestion, bile acids act as signaling and antimicrobial compounds, shaping gut microbiota composition and reinforcing colonization resistance (30–33). Pathogens like *S. flexneri* have evolved strategies to resist bile acid stress, including efflux pumps, membrane remodeling, and biofilm formation (25). Previous studies have shown that DCA promotes *S. flexneri* aggregation and biofilm formation (34–36) and modulates virulence factor expression and epithelial adherence (37–39). However, the role of LCA, another predominant colonic bile acid, remains unexplored.

In this study, we investigated how LCA influences *S. flexneri* during extracellular growth and its early interactions with epithelial cells. We demonstrate that LCA triggers T3SS-dependent aggregation and that these aggregates retain the capacity to invade epithelial cells. Together, these findings identify LCA as a luminal cue that drives the formation of invasion-competent aggregates, suggesting a potential role for aggregates at the interface between extracellular colonization and epithelial invasion.

## RESULTS

### Lithocholic acid promotes aggregation in *S. flexneri*

**Fig 1:**
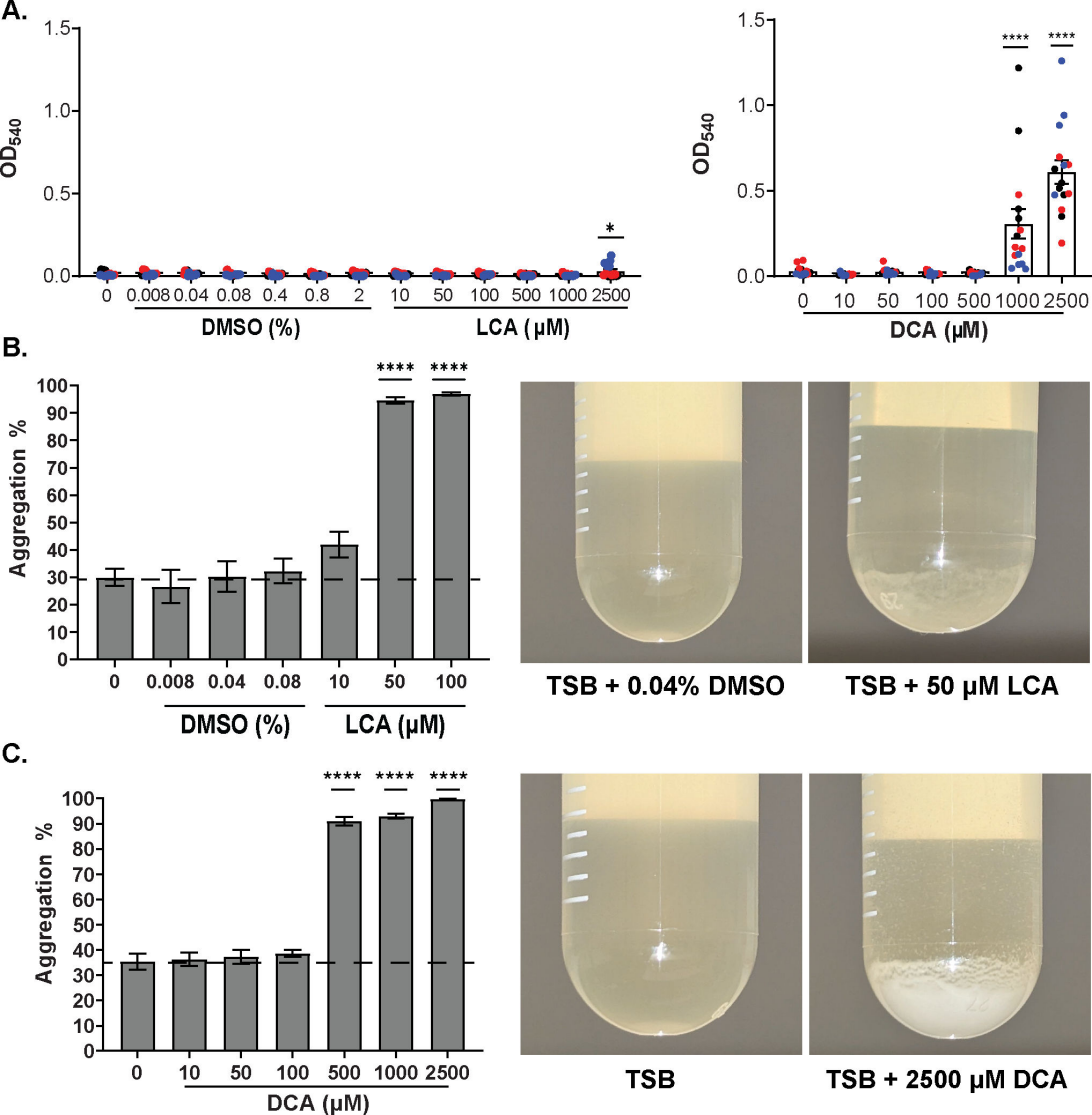
LCA induces robust aggregation in standing *S. flexneri* cultures. (**A**) Crystal violet staining of biofilm after 24-h growth (left) across increasing LCA concentrations paired with its matched dimethyl sulfoxide (DMSO) control and (right) increasing DCA concentrations. Graphs show mean ± SEM; each color represents an independent biological repeat (*n* = 3) (**B**). Sedimentation assay of *S. flexneri* in TSB containing increasing concentrations of LCA and vehicle (DMSO). (**C**) Sedimentation assay of *S. flexneri* grown in TSB with increasing concentrations of DCA. Aggregation % was calculated as (OD_600_ of total − OD_600_ of planktonic)/OD_600_ total × 100). OD_600_ total represents the fully resuspended culture, and OD_600_ planktonic represents the surface-collected fraction. The dashed line indicates basal bile acid-independent aggregation. Representative culture tubes are shown with indicated bile acid concentrations. Statistics (**A**) Left: Two-way ANOVA with Tukey’s multiple comparison test; Right: One-way ANOVA with Dunnett’s multiple comparison test; (**B**) and (**C**) Two-way ANOVA with Tukey’s multiple comparison test. Each LCA concentration in (**A**) and (**B**) was compared to the corresponding DMSO control. Each DCA concentration in (**A**) and (**C**) was compared to the TSB control (0 µM). **, *P* < 0.01; ****, *P* < 0.0001.

Deoxycholic acid (DCA) and lithocholic acid (LCA) are the two predominant secondary bile acids in the colon (29). While DCA has been previously shown to promote biofilm formation and aggregation under static growth conditions (34–36), the impact of LCA on *S. flexneri* community behavior had not been examined. We first assessed biofilm formation in Tryptic Soy Broth supplemented with increasing concentrations of LCA (10–2,500 μM) using crystal violet staining. Across this range, LCA had no measurable effect on biofilm development on polystyrene surfaces, in contrast to DCA (Fig. 1A). We next tested the effect of LCA on *S. flexneri* aggregation. LCA induced readily visible aggregation at concentrations as low as 50 μM, forming clumps at the bottom of culture tubes after 24 h of static incubation at 37°C (Fig. 1B). However, DCA induced aggregation only at substantially higher concentrations (500–2,500 μM) (Fig. 1C), indicating that LCA is drastically more potent at inducing aggregation. To validate OD_600_ as a measure of aggregation, we independently quantified aggregation using CFUs. Both methods yielded similar aggregation ratios (Fig. S1), confirming OD_600_ as a reliable readout.

Because bile acids can exhibit antimicrobial activity, we next asked whether LCA affects *S. flexneri* growth under these conditions. At 50 μM, LCA did not alter exponential growth, but LCA-treated cultures reached the stationary phase at lower OD_600_ and CFU compared to control conditions (Fig. S2A), while DCA showed no effect at this concentration (Fig. S2B). Together, these findings identify LCA as a potent inducer of *S. flexneri* aggregation at physiological concentrations.

### Dissecting the genetic basis of LCA-induced *S. flexneri* aggregation

**Fig 2:**
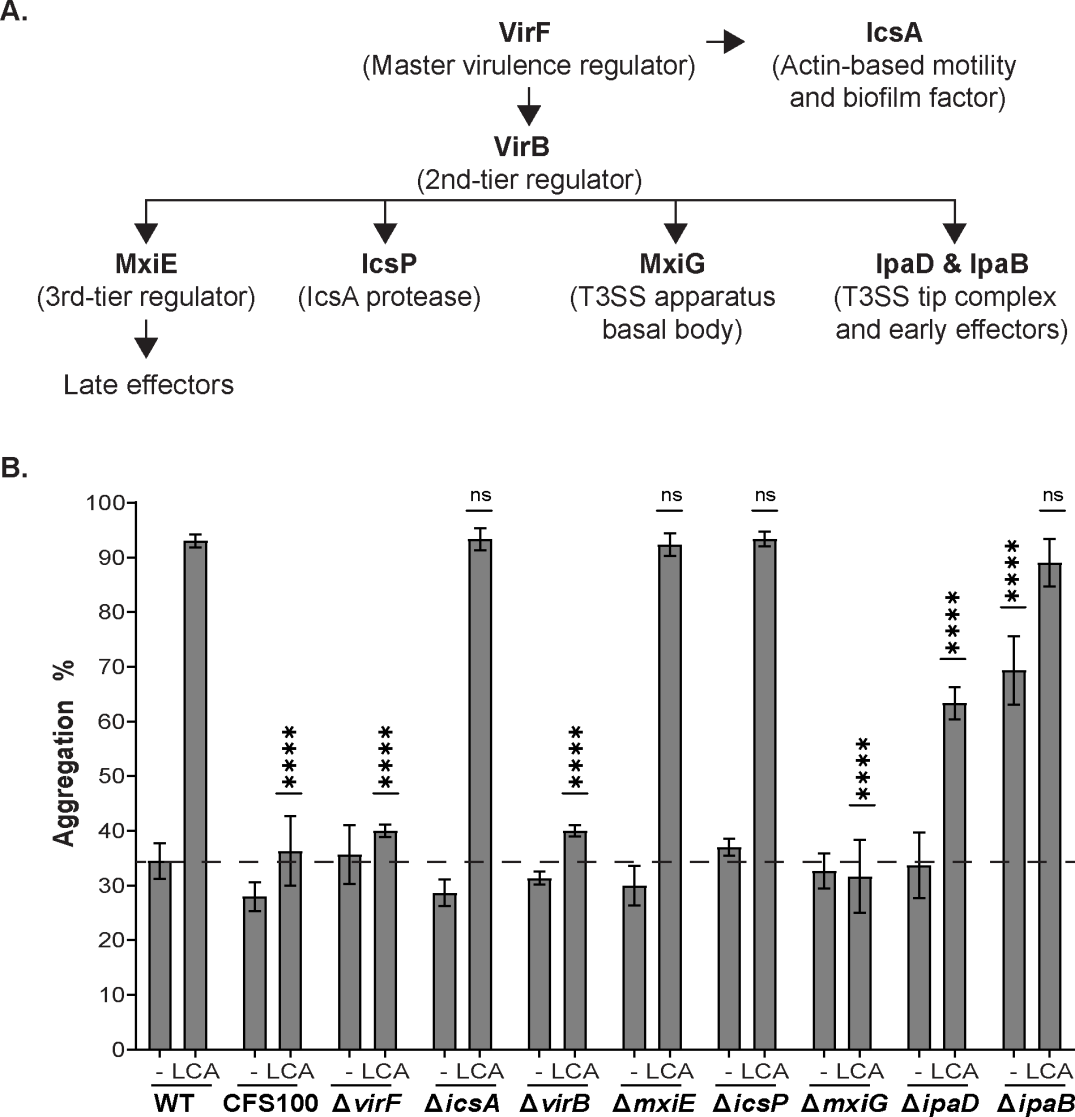
LCA induces *S. flexneri* aggregation via the VirF/VirB regulation and T3SS/lpaD-dependent mechanisms. (**A**) Schematic of plasmid-encoded regulatory cascades and T3SS-associated genes tested in this study. Bold labels indicate mutated genes; arrows denote established regulatory relationships. (**B**) Sedimentation assay of *S. flexneri* mutant strains grown in TSB supplemented with 50 µM LCA or DMSO vehicle (−). The graph shows mean ± SD from three biological repeats. The dashed line indicates basal bile acid-independent aggregation. Statistics: Two-way ANOVA with Tukey’s multiple comparison test relative to the corresponding WT control (WT/− vs Mutant/− and WT/LCA vs Mutant/LCA). ****, *P* < 0.0001; ns, not significant (*P* > 0.05).

*S. flexneri* harbors a 218-kb plasmid that encodes essential factors for virulence, including transcriptional regulators VirF, VirB, and MxiE, the T3SS, and IcsA (40–43) (Fig. 2A). To determine whether plasmid-encoded components mediate aggregation in response to LCA, we tested the plasmid-cured strain CFS100 (44). CFS100 failed to aggregate in the presence of 50 or 100 μM LCA (Fig. 2B; Fig. S3), indicating that LCA-induced aggregation requires plasmid-encoded factors.

**Fig 3:**
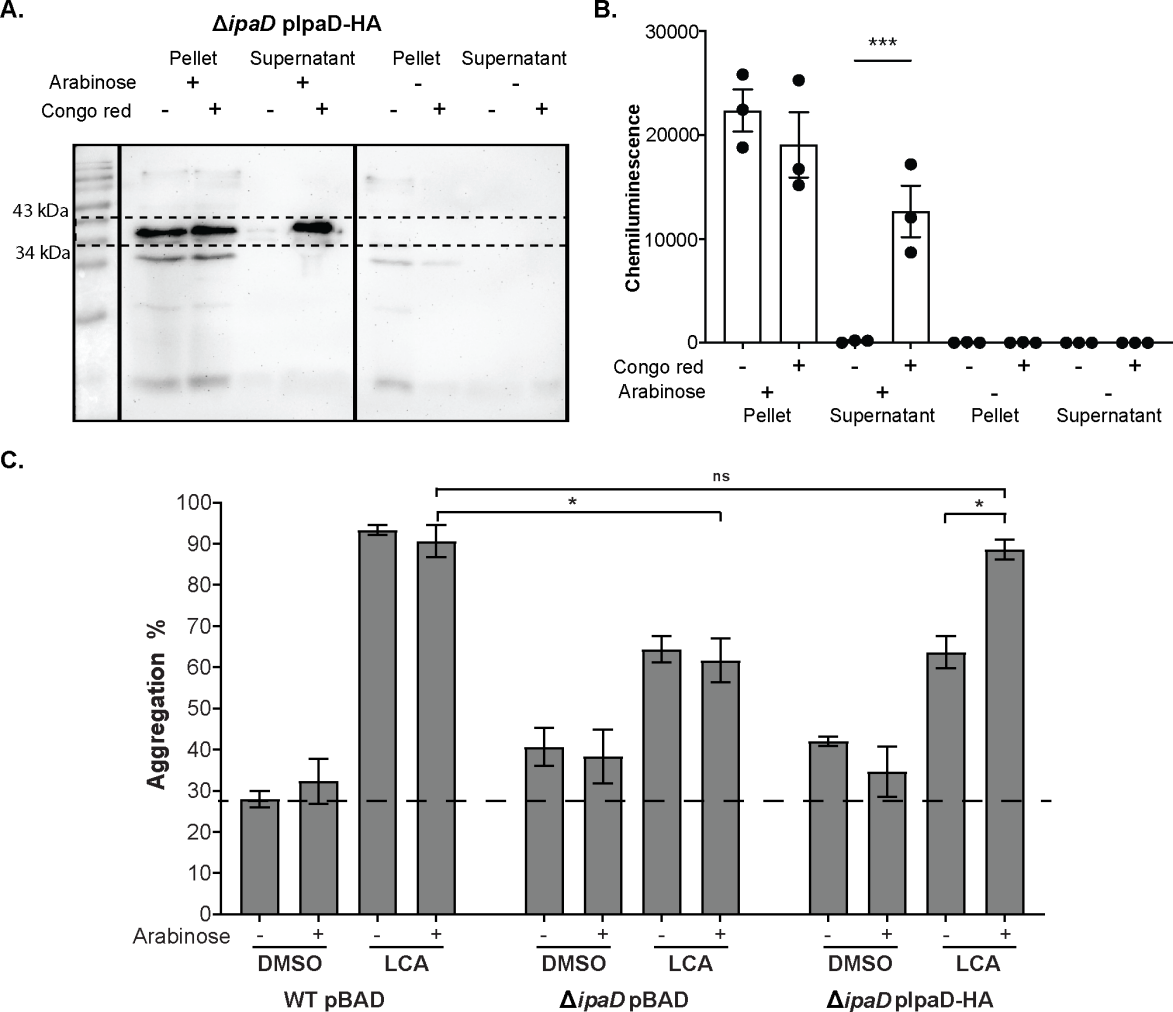
lpaD-HA expression rescues Δ*ipaD* defect in LCA-induced aggregation. (**A**) Representative Western blot showing induced expression with 0.1% (wt/vol) arabinose and Congo red-induced secretion of lpaD-HA (dashed box: 34–43 kDa) in Δ*ipaD* plpaD-HA strain (**B**). Quantification of lpaD-HA bands by Fiji. The graph shows mean ± SD from three biological repeats. Statistics: Three-way ANOVA. ***, *P* < 0.001. (**C**) Sedimentation assay for LCA-induced aggregation using strains harboring pBAD18 (pBAD) and plpaD-HA. The graph shows mean ± SD from three biological repeats. The dashed line indicates basal bile acid-independent aggregation. Statistics: Three-way ANOVA with Tukey’s multiple comparison test. *, *P* < 0.05; ns, not significant (*P* > 0.05).

We next performed a systematic mutational analysis (Fig. 2A) to identify specific genetic determinants. Deletion of *virF*, encoding the master virulence regulator (45), abolished LCA-induced aggregation (Δ*virF*, Fig. 2B; Fig. S3). Among VirF-regulated genes, deletion of *virB* also prevented aggregation, whereas deletion of *icsA* had no effect (Δ*virB* and Δ*icsA,* Fig. 2B; Fig. S3). Further analysis of the VirB regulon revealed that the deletion of *icsP* or *mxiE* did not impair aggregation (Δ*icsP* and Δ*mxiE*, Fig. 2B; Fig. S3). In contrast, deletion of *mxiG*, which encodes a core component of the T3SS basal body (46, 47), completely abolished LCA-induced aggregation (Δ*mxiG*, Fig. 2B; Fig. S3), implicating the T3SS in this process. We then focused on the T3SS tip complex components, IpaD and IpaB effectors (48–50). The Δ*ipaD* mutant, which also lacks IpaB at the T3SS needle tip (49), exhibited a significant reduction in LCA-induced aggregation (Fig. 2B; Fig. S3), identifying IpaD as a key contributing factor. To confirm the specific contribution of IpaD, we constructed an arabinose-inducible *ipaD-HA* expression system and verified its expression and secretion using a Congo red-induced secretion assay (Fig. 3A and B). Arabinose induction restored aggregation in the Δ*ipaD* strain (Fig. 3C), confirming the functional role of IpaD in LCA-induced aggregation. By contrast, the Δ*ipaB* mutant aggregated normally in the presence of LCA but interestingly showed a hyper-aggregation phenotype in media lacking LCA (Fig. 2B; Fig. S3). This suggests IpaB may negatively regulate basal aggregation when bile acid signals are absent. Based on the hyper-aggregation phenotype of the Δ*ipaB* mutant in the absence of LCA (Fig. 2B; Fig. S3) and the previously reported IpaB oversecretion in Δ*ipaD* (49), we tested the hypothesis that IpaB may contribute to defective Δ*ipaD* aggregation. However, a Δ*ipaD* Δ*ipaB* double mutant remained defective in LCA-induced aggregation (Fig. S4), ruling out any IpaB role in the Δ*ipaD* aggregation defect. Consistent with a protein-dependent mechanism, proteinase K treatment eliminated LCA-induced aggregation (Fig. S5), supporting the involvement of proteinaceous components, including the T3SS and IpaD.

To investigate whether IpaD directly interacts with LCA, we performed *in silico* docking, which identified four potential LCA-contact residues (Fig. S6A). While the accessibility of these residues in the assembled pentameric tip complex remains to be determined (48, 51), alanine substitutions at these positions did not alter LCA-induced aggregation relative to IpaD-HA complementation (Fig. S6B), suggesting that LCA binding may involve multiple residues or an indirect allosteric contribution of IpaD to aggregation. Together, these findings demonstrate that LCA promotes *S. flexneri* aggregation through a virulence plasmid-encoded, T3SS-dependent mechanism that requires the tip complex protein and effector, IpaD.

### IpaD is required for the initiation of LCA-induced aggregation

**Fig 4:**
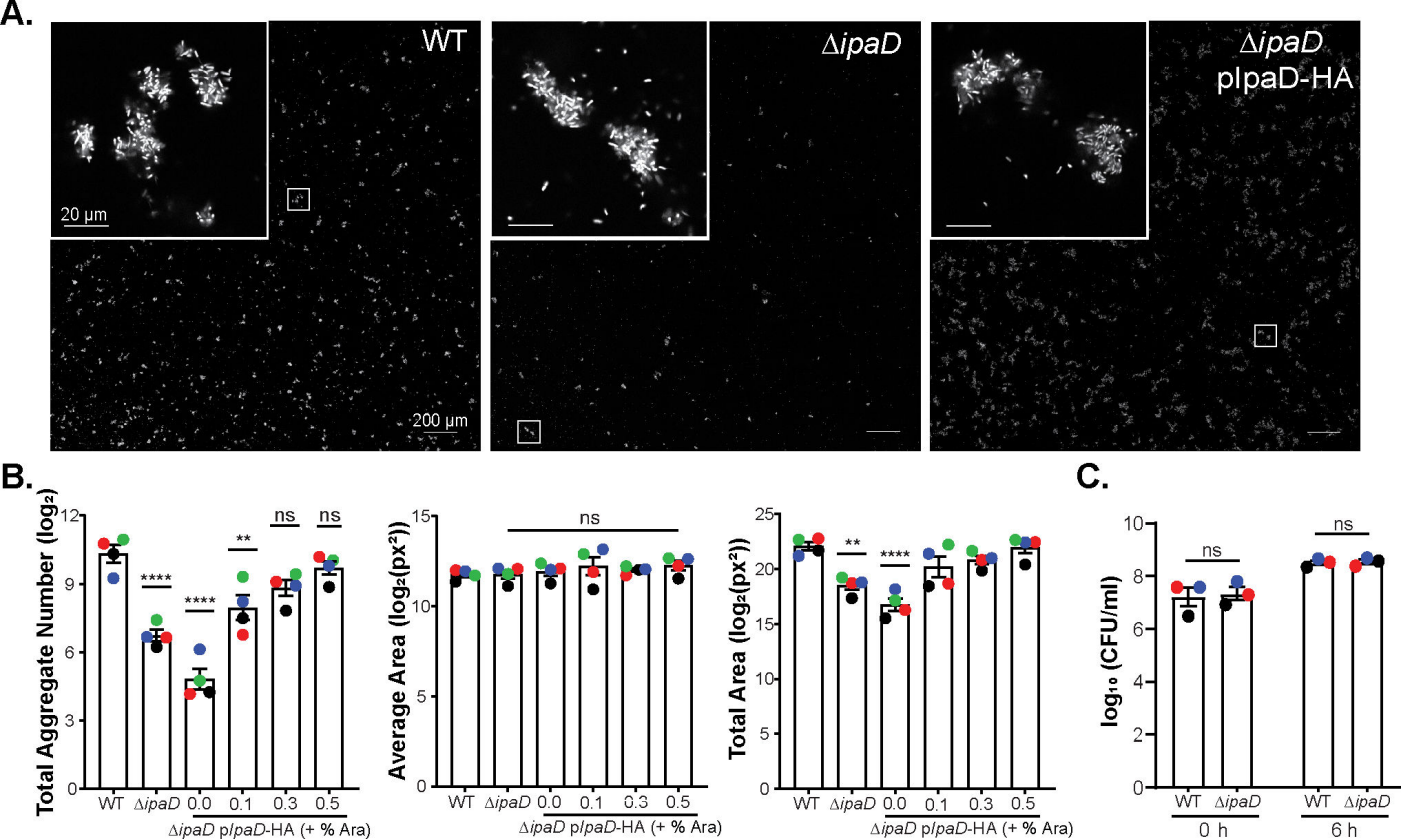
lpaD determines the number of LCA-induced aggregates at 6 h. (**A**) Representative widefield images (40× objective) of sedimented aggregates from WT, Δ*ipaD*, and Δ*ipaD* plpaD-HA (+0.5% arabinose) strains after 6 h static growth with 50 µM LCA. White boxes indicate inset images, showing individual aggregates at higher magnification. All images were acquired using the same exposure settings. (**B**) Quantification of total number, average area, and total area for aggregates of WT, Δ*ipaD*, and Δ*ipaD* plpaD-HA that was incubated in the presence of increasing arabinose (Ara) levels for 6 h with 50 µM LCA. Aggregates were quantified using a minimum area threshold of 1,000 px^2^ (∼10 bacteria). Graphs show mean ± SD from four biological repeats, indicated by different colors. (**C**) CFU enumeration of WT and Δ*ipaD* at 0 and 6 h under static conditions with 50 µM LCA. Graphs show mean ± SD from three biological repeats, indicated by different colors. Statistics: (**B**) One-way ANOVA with Tukey’s multiple comparison test, (**C**) Two-way ANOVA with Tukey’s multiple comparison test . **, *P* < 0.01; ****, *P* < 0.0001; ns, not significant (*P* > 0.05).

To further elucidate the role of IpaD in LCA-induced aggregation, we performed imaging of sedimented aggregates of WT and Δ*ipaD* strains. Unlike the Δ*ipaD* pIpaD-HA strain*,* sedimented WT and Δ*ipaD* aggregates at 24 h formed large, continuous structures with poorly defined boundaries, which precluded reliable size quantification of individual aggregates (Fig. S7A). We therefore analyzed the size of aggregates formed by the Δ*ipaD* pIpaD-HA strain, with or without arabinose-mediated induction of IpaD-HA expression. SYTO9-stained aggregates were imaged and their projected areas quantified. In the absence of arabinose, corresponding to the Δ*ipaD* mutant, sedimented aggregates were significantly smaller, whereas induction of IpaD-HA expression resulted in the formation of larger aggregates (Fig. S7B and C), suggesting that IpaD influences aggregate size. To better understand the role of IpaD in LCA-induced aggregation, we analyzed WT, Δ*ipaD*, and Δ*ipaD* pIpaD-HA aggregates at 6 h, prior to extensive sedimentation and community reorganization observed at 24 h. The Δ*ipaD* strain formed fewer aggregates than WT (widefield images, Fig. 4A; left graph, Fig. 4B) dramatically, with a corresponding reduction in total aggregate area (right graph, Fig. 4B), both measured using a minimum aggregate size threshold of 1,000 px^2^ (~10 bacteria). Consistent results were observed across higher minimum size thresholds of 2,000 px^2^ (~20 bacteria) and 4,000 px^2^ (~40 bacteria) (Fig. S8). These thresholds define the minimum number of bacteria required to constitute an aggregate. Induction of IpaD-HA expression in Δ*ipaD* rescued aggregate number and total aggregate area (widefield images, Fig. 4A; left and right graphs, Fig. 4B; left and right graphs, Fig. S8A and B), confirming the specific contribution of IpaD to this process. Despite the reduction in aggregate number, mean aggregate size remained comparable between WT and Δ*ipaD* (insets in widefield images, Fig. 4A; middle graph, Fig. 4B; middle graphs, Fig. S8A and B), suggesting that IpaD specifically drives the frequency of aggregation events at this early stage rather than individual aggregate growth. To rule out growth rate differences as a confounding factor, we enumerated CFUs at 0 and 6 h for WT and Δ*ipaD* under the same static conditions. WT and Δ*ipaD* reached comparable CFU counts at 6 h (Fig. 4C), indicating that the aggregation defect of Δ*ipaD* cannot be attributed to growth alterations. Collectively, these data demonstrate that IpaD is essential for aggregate initiation, while it may also influence aggregate size at later time points, supporting its central role in the T3SS-dependent LCA-induced aggregation mechanism.

### Interactions between LCA-induced *S. flexneri* aggregates and epithelial cells

**Fig 5:**
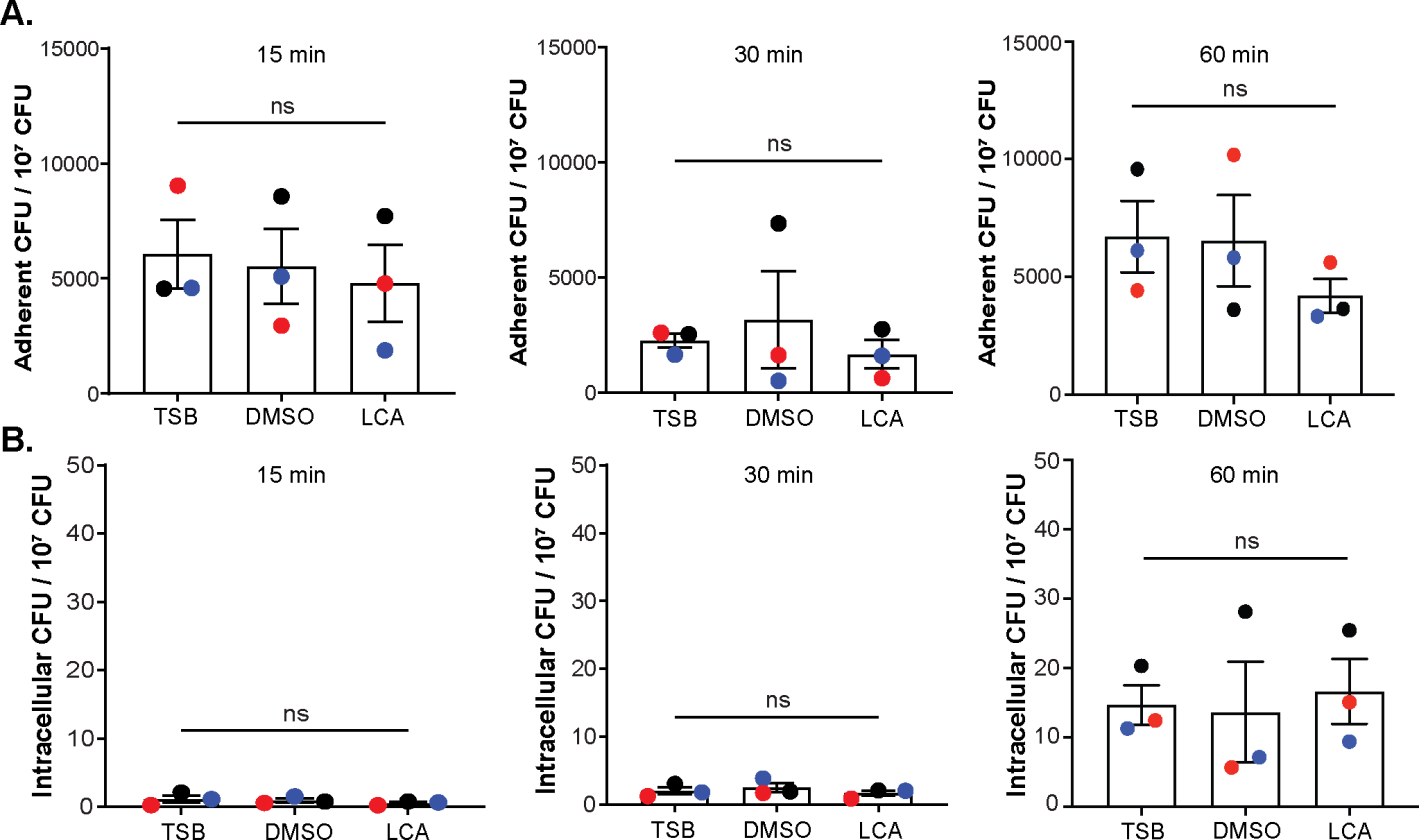
LCA-induced *S. flexneri* aggregates adhere to and invade HT-29 cells. (**A**) Adhesion to HT-29 cells at an MOI of 200 by 24 h non-aggregating controls grown in TSB, TSB+ vehicle (DMSO), and 24 h LCA-induced aggregates (LCA) at 15, 30, and 60 min post-infection. (**B**) Invasion of HT-29 cells at an MOI of 200 by 24 h non-aggregating controls grown in TSB, TSB+ vehicle (DMSO), and 24 h LCA-induced aggregates (LCA) at 15, 30, and 60 min post-infection. Graphs show mean ± SD from three biological repeats, indicated by different colors. Statistics: One-way ANOVA with Tukey’s multiple comparison test. ns, not significant (*P* > 0.05).

**Fig 6:**
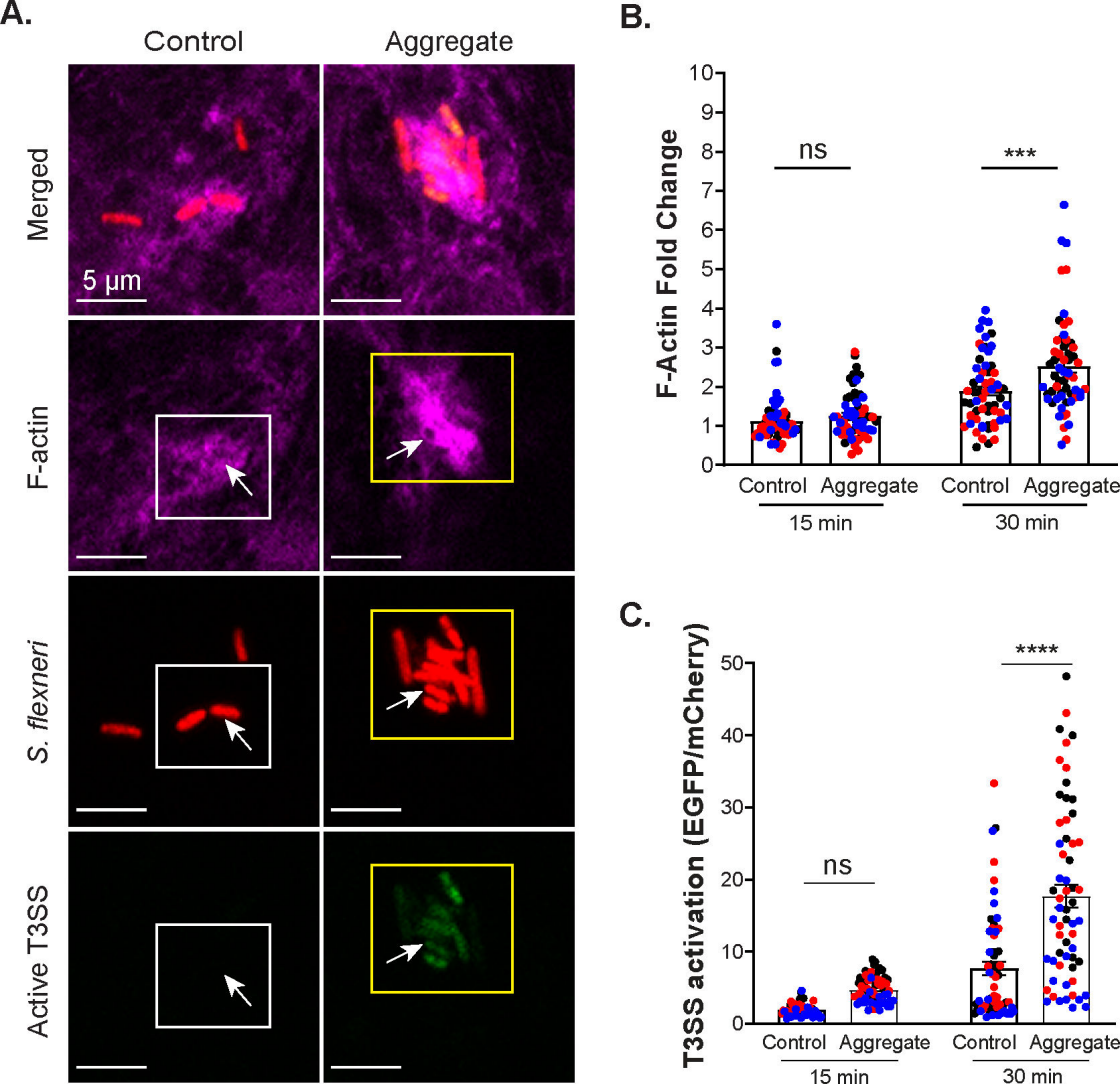
LCA-induced aggregates confer strong actin polymerization and display increased T3SS activity during cell interactions at 30 min. (**A**) Representative images of 30 min infections at an MOI of 20 with *S. flexneri* grown in TSB containing 0.04% DMSO (Control) or 50 µM LCA (Aggregate) in HT-29 monolayers. Merged image, maximum intensity projection; representative single Z-plane, F-actin (magenta); representative maximum intensity projections, *S. flexneri* (mCherry) and Active T3SS (EGFP). White arrows indicate vacuole-associated bacteria; white boxes denote bacteria lacking T3SS activity; yellow boxes denote T3SS-active bacteria (EGFP-positive). Scale bar, 5 µm (B). Quantification of F-actin focus intensity via normalization to uninfected cell regions. (**C**) Quantification of EGFP fluorescence intensity via normalization to mCherry fluorescence intensity per infection focus, reporting T3SS activity per bacterial signal. Graphs show mean ± SEM from three biological repeats, indicated by different colors. Each point represents a single measurement. Statistics (B) and (**C**): Two-way ANOVA with Tukey’s multiple comparison test. ***, *P* < 0.001; ****, *P* < 0.0001; ns, not significant (*P* > 0.05).

Given that LCA induces *S. flexneri* aggregation through a T3SS/IpaD-dependent mechanism, we next characterized the interaction between 24 h aggregates and colonic epithelial cells during the early phase of infection. To assess adhesion and invasion, we performed gentamicin protection assays using HT-29 and Caco-2 cells and quantified adherent and invasive bacteria at 15, 30, and 60 min, each followed by 1-h gentamicin treatment. LCA-induced aggregates produced adherent and invasive burdens comparable to non-aggregating controls (LCA-independent bacterial pellets) in both HT-29 (Fig. 5) and Caco-2 (Fig. S9) cells, indicating that aggregates retain adhesive and invasive capacity. To compare early host-pathogen interaction dynamics, we used a T3SS reporter plasmid that constitutively expresses mCherry to label bacteria and produces EGFP upon active T3SS secretion, whereby secretion of early effectors releases MxiE to drive EGFP transcription in *S. flexneri* (52). Reporter-expressing non-aggregating bacteria and LCA-induced aggregates were applied to confluent HT-29 monolayers at MOI 20 and 200 and fixed at 15 and 30 min post-infection, time points selected to capture early aggregate-specific interactions. At an MOI of 20, infections with LCA-induced aggregates triggered the formation of F-actin foci at 15 and 30 min, significantly stronger than in infections with non-aggregating controls at 30 min (Fig. 6A and B). At an MOI of 200, LCA-induced aggregates elicited greater F-actin reorganization than the non-aggregating control at 15 min, with comparable responses observed between conditions at 30 min in HT-29 cells (Fig. S10A). In Caco-2 cells at an MOI of 200, LCA-induced aggregates and the non-aggregating control triggered comparable F-actin reorganization at both time points (Fig. S10B). These findings suggest that LCA-induced aggregates manipulate the host cytoskeleton via active secretion of T3SS effectors that modulate actin reorganization dynamics during invasion (53, 54). Consistently, LCA-induced aggregates exhibited similar T3SS activity to the non-aggregating control at 15 min and higher T3SS activity per bacterial signal by 30 min at MOI 20, as shown by elevated EGFP/mCherry ratios (EGFP, yellow box vs white box, Fig. 6A and C), indicating that the difference reflects T3SS activity normalized to bacterial signal rather than an effect of aggregate size. A similar pattern of elevated T3SS activation was observed at an MOI of 200 in HT-29 (Fig. S10A) and Caco-2 cells (Fig. S10B). Moreover, aggregate-associated bacteria were frequently observed within actin-encased vacuoles (white arrows, Fig. 6A; Fig. S11), an intermediate stage preceding vacuolar escape (55). Together, these data show that LCA-induced aggregates remain invasion-competent and initiate epithelial interactions characterized by regular actin remodeling and elevated T3SS activation, supporting the notion that aggregate-mediated invasion represents a new mode of epithelial entry.

## DISCUSSION

### A specific role for LCA in *S. flexneri* aggregation

The secondary bile acids DCA and LCA comprise the majority of the colonic bile acid pool, as evidenced by fecal bile acid composition (DCA 34% and LCA 29%) (29). LCA, derived from primary bile acid chenodeoxycholic acid by gut microbiota (29), modulates bacterial physiology and virulence factors in Gram-positive pathogens. For example, it downregulates toxin expression, toxin activity, and germination in *Clostridioides difficile* and promotes biofilm formation and altered morphology in vancomycin-resistant *Enterococcus faecium* (56–58). Whether and how LCA influences Gram-negative enteric pathogens remains largely unexplored. We identify LCA as a signal that drives *S. flexneri* aggregation at physiological concentrations (50–100 μM) (59, 60) (Fig. 1B), making it far more potent than DCA, which requires substantially higher concentrations to induce aggregation (Fig. 1C). LCA does not promote *S. flexneri* biofilm formation on polystyrene surfaces, in contrast to DCA, which increases surface-attached biofilm biomass under the same conditions (Fig. 1A), as shown previously (34–36). LCA can exhibit antimicrobial activity (61), yet it did not significantly affect *S. flexneri* growth rate under our experimental conditions, as confirmed by OD_600_ and CFU measurements (Fig. S2). Therefore, under these *in vitro* conditions, LCA induces a mode of community assembly distinct from canonical biofilm growth in the Gram-negative pathogen *S. flexneri*. Given that DCA and LCA are naturally co-present in the colon, how their combined presence influences *S. flexneri* community behavior represents an interesting direction for future investigation.

### An extracellular role for IpaD in T3SS-dependent LCA-induced aggregation

Bacterial aggregation can arise through different biophysical mechanisms shaped by environmental constraints on motion or by the presence of bacterial and host-derived polymeric structures (62). Although the molecular mechanism by which LCA promotes aggregation remains unknown, we showed that LCA-induced aggregation requires several virulence plasmid-encoded factors, including the transcriptional regulators VirF and VirB, the T3SS basal body component MxiG, and the T3SS tip protein IpaD (Fig. 2; Fig. S3), revealing a previously unrecognized, T3SS-dependent mode of community formation triggered by a host-derived metabolite.

Our data identify IpaD as a key contributor to this process. The Δ*ipaD* mutant is impaired in aggregation at 24 h (Fig. 2B; Fig. S3; Fig. 3C), and induction of IpaD expression increases aggregate size (Fig. S7B and C) at this time point. The imaging of 6-h aggregates revealed that Δ*ipaD* generates approximately 10-fold fewer aggregates compared to WT, a phenotype that is rescued by the inducible expression of IpaD-HA (Fig. 4; Fig. S8). These data suggest that IpaD is necessary to initiate LCA-induced aggregation at early stages, while it may also influence aggregate size at later time points. IpaD is not the sole determinant of aggregation: the Δ*virF*, Δ*virB*, and Δ*mxiG* mutants are completely defective (Fig. 2B; Fig. S3). This is consistent with the essential role of MxiG in T3SS apparatus assembly, which is required for delivery of effectors, including IpaD and IpaB, to the needle tip (46, 63, 64). This suggests that IpaD functions in concert with other T3SS structural components or early effectors. Late effectors appear dispensable, as the Δ*mxiE* mutant aggregates normally (Fig. 2B; Fig. S3).

IpaD is a T3SS effector essential for invasion of epithelial cells (65). It localizes to the tip of the T3SS needle (48), and exposure to DCA allows IpaD to recruit IpaB to the T3SS tip complex (49). In association with IpaB, IpaD also regulates T3SS-dependent secretion by repressing premature effector release (66). We showed that LCA-induced aggregation does not require IpaB, as the Δ*ipaB* strain aggregates normally in the presence of LCA but displays a hyper-aggregation phenotype in the absence of LCA (Fig. 2B; Fig. S3). These findings suggest that IpaB may function as a negative regulator of bile acid-independent aggregation. Furthermore, the Δ*ipaD* Δ*ipaB* double mutant remained defective in LCA-induced aggregation (Fig. S4), confirming that the aggregation defect of Δ*ipaD* is independent of IpaB. We propose that IpaD supports initiation of aggregation, while additional T3SS-related factors support aggregate growth in the presence of LCA. Future studies will define the molecular mechanism by which IpaD drives LCA-induced aggregation initiation.

Docking simulations identified four candidate IpaD residues potentially involved in LCA interaction (Fig. S6A). Alanine substitutions at these positions do not impair LCA-induced aggregation (Fig. S6B). However, given the conservative nature of alanine substitutions, these findings do not rule out the involvement of these residues in LCA-responsive IpaD activity. While direct binding remains to be demonstrated, current evidence suggests that LCA may modulate IpaD function indirectly, possibly by altering its expression, secretion, or interactions with other T3SS components. Taken together, these findings expand the functional repertoire of IpaD beyond its canonical role in invasion, revealing an extracellular, LCA-responsive function in *S. flexneri* aggregation.

### Implications of LCA-induced aggregation for *S. flexneri*-host interactions

Bacterial aggregates are increasingly recognized as pathogenic communities across various infectious diseases (67). These communities can exhibit behaviors distinct from those of planktonic bacteria, particularly during early host-cell interactions. For example, *Streptococcus pyogenes* aggregates, mediated by surface proteins, show enhanced adherence to host cells relative to non-aggregating *S. pyogenes* (68). Similarly, *Pseudomonas aeruginosa* aggregates promote invasion by facilitating their internalization by MDCK cells (69), while *Listeria monocytogenes* aggregates enhance both adhesion and InlB-dependent invasion in HeLa cells (70). These examples illustrate the diverse ways in which aggregates can participate in epithelial invasion. Consistent with invasion-competent aggregates, our gentamicin protection assays show that 24 h LCA-induced *S. flexneri* aggregates adhere to and invade epithelial HT-29 and Caco-2 cells at rates comparable to non-aggregating bacteria (Fig. 5; Fig. S9). In addition, imaging analyses of initial interactions with epithelial cells revealed that LCA-induced aggregates elicit regular actin reorganization and elevated T3SS activation at 30 min upon host cell contact (Fig. 6; Fig. S10). These findings suggest that, in addition to the well-established invasion mode driven by exponentially growing, non-aggregating *S. flexneri*, LCA-induced aggregate-mediated invasion constitutes an alternative mode for epithelial entry.

When considered with *in vivo* observations of *S. flexneri* aggregates (23, 24), our data support the hypothesis that LCA-induced aggregation serves as a bridge between extracellular colonization and epithelial invasion during *S. flexneri* infection. This mode of entry raises important questions regarding how aggregate-driven invasion affects subsequent intracellular infection dynamics and cellular responses.

Beyond epithelial interactions, LCA-induced aggregates may also confer ecological advantages in the intestinal lumen. First, T3SS/IpaD-dependent aggregation may cluster virulent bacteria in ways that spatially separate them from T3SS-defective subpopulations. Although this possibility remains unexplored, such an organization could protect T3SS-proficient cells from luminal factors, such as proteases, that can disrupt T3SS function, thereby helping preserve effector delivery. Second, aggregates may enhance resistance to environmental stressors yet to be characterized. Unlike *S. sonnei, S. flexneri* lacks both a T6SS (71) and a capsule (72). We propose that bile acid-driven community formation may help compensate for these deficiencies: DCA primarily promotes biofilm formation (34, 35), whereas LCA drives T3SS/IpaD-dependent aggregation. Together, these distinct bile acid-linked mechanisms may reinforce *S. flexneri* survival in the lumen while generating invasion-competent clusters. More broadly, LCA-induced, T3SS-dependent aggregation may reflect a general adaptation among Gram-negative enteric pathogens and a potential target for therapeutic intervention.

## MATERIALS AND METHODS

### Bacterial strains, bile acid media preparation, and cell culture

*Shigella flexneri* strain 2457T, provided by Dr. Benjamin Koestler (Western Michigan University), was used as the model pathogen, and *Escherichia coli* DH5α was used for cloning (Table 1). Bacterial stocks were maintained at −80°C in Tryptic Soy Broth (TSB; Sigma-Aldrich, 22092-500G) supplemented with 20% glycerol. Frozen *S. flexneri* stocks were streaked onto Lysogeny Broth (LB; Fisher, BP1426-2) agar plates containing 10 µg/mL Congo red (Sigma-Aldrich, SDC6277) and incubated at 30°C or 37°C overnight to obtain isolated colonies. When required, culture media were supplemented with ampicillin (100 µg/mL; Sigma-Aldrich Fine Chemicals, NC1004831), kanamycin (30 µg/mL; Thermo Scientific, AC450810100), or chloramphenicol (10 µg/mL; Sigma-Aldrich Fine Chemicals, 501786841).

**TABLE 1:** Bacterial strains used in this study

Strain	Description	Source/reference
*S. flexneri* 2457T	Wild type	Dr. Benjamin Koestler, Western Michigan University/(42)
CFS100	*pINV*-cured 2457T	Dr. Benjamin Koestler, Western Michigan University
*S. flexneri virF*::*kanR*	Gene replacement strain	This study
*S. flexneri virB*::*kanR*	Gene replacement strain	This study
*S. flexneri mxiE*::*kanR*	Gene replacement strain	This study
*S. flexneri mxiG*::*kanR*	Gene replacement strain	This study
*S. flexneri icsA*::*kanR*	Gene replacement strain	This study
*S. flexneri icsP*::*kanR*	Gene replacement strain	This study
*S. flexneri ipaD*::*kanR*	Gene replacement strain	This study
*S. flexneri* Δ*ipaD*	Clean deletion mutant	This study
*S. flexneri ipaB*::*kanR*	Gene replacement strain	This study
*S. flexneri* Δ*ipaD ipaB*::*kanR*	Gene replacement in Δ*ipaD*	This study
*E. coli* DH5α	Cloning strain	(35)

All bile acid solutions were prepared fresh for each experiment. Sodium deoxycholate (DCA; Sigma-Aldrich, D6750-25G, ≥97% purity) was dissolved directly in TSB and sterilized using 0.22 µm filter units (MilliporeSigma, SLGSR33SS). Lithocholic acid (Sigma-Aldrich, L6250-10G, ≥95% purity) was dissolved in dimethyl sulfoxide (Fisher Scientific, 317275500ML) and sterilized using DMSO-compatible 0.22 µm filter units (CellTreat, 229757).

The human colorectal epithelial cell lines HT-29 (HTB-38; ATCC) and Caco-2 (a gift from Dr. M. Ammar Zafar, Emory University) were maintained at 37°C with 5% CO_2_. HT-29 cells were cultured in McCoy’s 5A medium (Gibco, 16600-108) supplemented with 10% heat-inactivated fetal bovine serum (FBS; Gibco, 26140-079). Caco-2 cells were cultured in Dulbecco’s Modified Eagle Medium (DMEM; Gibco, 11995-073) supplemented with 20% heat-inactivated FBS. Upon reaching 70%–90% confluency, monolayers of both cell lines were washed three times with phosphate-buffered saline (DPBS; Gibco, 14190-250) and detached with 0.25% trypsin-EDTA. Cells were routinely passaged at 1:5 or 1:10 dilutions and used between passages 4 and 19, and confirmed mycoplasma-free by the Cell Engineering Shared Resource core facility at Wake Forest University School of Medicine.

### Bacterial growth assays

For growth curve experiments, bile acid-supplemented TSB was dispensed into 96-well plates after inoculation with bacterial colony suspensions, and optical density at 600 nm (OD_600_) was recorded at 37°C hourly using a microplate reader (Synergy H1, BioTek). Colony-forming units (CFUs) were enumerated at 4 h and 24 h to validate OD_600_ measurements. To assess whether growth rate differences account for aggregation phenotype differences between *S. flexneri* WT and Δ*ipaD* strains, CFUs were enumerated at 0 and 6 h from cultures grown under the same static conditions as the sedimentation assay (see below). Samples were serially diluted and plated on LB agar supplemented with Congo red to enumerate CFUs.

### Construction of *S. flexneri* mutants

Mutant strains were generated using λ-red recombineering (73) (Table 1). PCR fragments were amplified from template plasmid pKD4 (Table 2), which carries a kanamycin-resistance cassette, using primers listed in Table 3. Each fragment contained 42 bp homology regions corresponding to sequences flanking the target gene. *S. flexneri* harboring pKD46 was grown at 30°C with arabinose induction (0.1% [wt/vol]) until the exponential phase (OD_600_ of 0.5–0.6). Electrocompetent cells were prepared and transformed with PCR products via electroporation (1.8 kV, 400 Ω, 25 µF). After overnight recovery growth, recombinants were selected on kanamycin plates and verified by colony PCR using confirmation primers located outside the deletion region (Table 3). To remove the kanamycin cassette, mutants were transformed with pCP20 (Table 2), serially passaged at 37°C, and screened for loss of both kanamycin and ampicillin resistance. Cassette excision was verified by PCR using confirmation primers (Table 3). For complementation, IpaD was cloned into pBAD18 (Table 2) with a C-terminal HA tag. The gene was amplified by PCR, digested with EcoRI-HF and SphI-HF, and ligated into CIP-treated pBAD18 digested with the same restriction enzymes. Constructs were verified by Sanger sequencing.

**TABLE 2:** Plasmids and constructs used in this study

Plasmid	Description (reference)	Source
pKD46	Carries λ-red recombination genes under arabinose induction (73)	NovoPro (V007013)
pKD4	Template plasmid carrying a kanamycin resistance (*kanR*) cassette flanked by FRT sites (73)	Dr. M. Ammar Zafar, Emory University
pCP20	Carries FLP recombinase for excision of the *kanR* cassette; temperature-sensitive origin (73)	Dr. M. Ammar Zafar, Emory University
pTSAR 3.4t	Fluorescent reporter for T3SS activity (52)	Addgene (#210256)
pBAD18	Arabinose inducible gene expression (74)	NovoPro (V001454)
pBAD18-IpaD-HA	Complementation plasmid expressing IpaD tagged with HA at the C-terminus	This study
pBAD18-IpaD(Q148A)-HA	Complementation plasmids expressing IpaD mutants with the indicated amino acid substitutions tagged with HA at the C-terminus	This study
pBAD18-IpaD(K151A)-HA		
pBAD18-IpaD(H155A)-HA		
pBAD18-IpaD(Y212A)-HA		

**TABLE 3:** Primers used in this study

Kanamycin cassette insertion confirmations		Sequence (5′ → 3′)
k-c-fwd		CGG TGC CCT GAA TGA ACT GC
k-c-rev		CAG TCA TAG CCG AAT AGC CT
*virF* mutation		Sequence (5′ → 3′)
Insertion fragment	virF fwd	ATG ATG GAT ATG GGA CAT AAA AAC AAA ATA GAT ATA AAG GTT TGT GTA GGC TGG AGC TGC TTC
	virF rev	ATA TAA GTA AAA TTT CTT TGG AGT TAT ACC ATA ATA TTC ATT CAT ATG AAT ATC CTC CTT AG
Confirmation	virF-k-c-fwd	GCT GCA TAA GCT CTT TCT TCA
	virF-k-c-rev	CCC GGC GGT GAA AAC GTT
*virB* mutation		Sequence (5′ → 3′)
Insertion fragment	virB fwd	ATG GTG GAT TTG TGC AAC GAC TTG TTA AGT ATA AAG GAA GGC TGT GTA GGC TGG AGC TGC TTC
	virB rev	TTA TGA AGA CGA TAG ATG GCG AGA AAT TAT ATC CCG AAT AGC CAT ATG AAT ATC CTC CTT AG
Confirmation	virB-k-c-fwd	CTA CCG TTG ACT ATC ATC AAC
	virB-k-c-rev	TAC CCC ACC GGC TGA ATT CCG
*icsA* mutation		Sequence (5′ → 3′)
Insertion fragment	icsA fwd	ATG AAT CAA ATT CAC AAA TTT TTT TGT AAT ATG ACC CAA TGT TGT GTA GGC TGG AGC TGC TTC
	icsA rev	TCA GAA GGT ATA TTT CAC ACC CAA AAT ACC TTG GGT GTC TCT CAT ATG AAT ATC CTC CTT AG
Confirmation	icsA-k-c-fwd	AGA CAC AGG TAA ATT TCT CCC GTT
	icsA-k-c-rev	CGC TTT CTA ATG CAA TTC CTG TGT
*icsP* mutation		Sequence (5′ → 3′)
Insertion fragment	icsP fwd	ATG GAC ATT TCA ACC AAA AAA GTA GAG TTC TCG ATG AAA TTA TGT GTA GGC TGG AGC TGC TTC
	icsP rev	TCA AAA AAT ATA CTT TAT ACC TGC GGA AGT GAG AAA ACC AAT CAT ATG AAT ATC CTC CTT AG
Confirmation	icsP-k-c-fwd	AGA GAA ATC TAT GGC CCC CGT TAC
	icsP-k-c-rev	AAG CGG TTC TTA TTT CTC CGG TCT
*mxiG* mutation		Sequence (5′ → 3′)
Insertion fragment	mxiG fwd	ATG TCT GAG GCA AAG AAC TCA AAT CTT GCA CCA TTC AGG TTA TGT GTA GGC TGG AGC TGC TTC
	mxiG rev	TCA CTT ATT TTT ATC TAA AAA AAA CCA GTG TTT ATC ATT CAA CAT ATG AAT ATC CTC CTT AG
Confirmation	mxiG-k-c-fwd	GGG TGG GAG GCT GTT GGA GCC TAT
	mxiG-k-c-rev	GCC AGC AAC TGT GGA TTC GAA GGA TTT TTA GCT
*mxiE* mutation		Sequence (5′ → 3′)
Insertion fragment	mxiE fwd	AAT GTA AGT AAT GCA CTG GCT ATG ATA CGA ATG ACT GAG TCA TGT GTA GGC TGG AGC TGC TTC
	mxiE rev	TTA ATA TTA AAT TTT TTC ATT TAT TTT TTT CAC TAA AAA AGT CAT ATG AAT ATC CTC CTT AG
Confirmation	mxiE-k-c-fwd	AAC CAG TTA ACG TTG AGC TTG
	mxiE-k-c-rev	GCA GCT TGT TTG CTA ACA ACA
*ipaD* mutation		Sequence (5′ → 3′)
Insertion fragment	ipaD fwd	ATG AAT ATA ACA ACT CTG ACT AAT AGT ATT TCC ACC TCA TCA TGT GTA GGC TGG AGC TGC TTC
	ipaD rev	TCA GAA ATG GAG AAA AAG TTT ATC TGT ATC TGT ACA TGA GCT CAT ATG AAT ATC CTC CTT AG
Confirmation	ipaD-k-c-fwd	ACA GCC AGT CAG ATT GCT GGT AAC
	ipaD-k-c-rev	TGT TGA TGA TGG TGA AGT TGC CTT
*ipaB* mutation		Sequence (5′ → 3′)
Insertion fragment	ipaB fwd	ATG CAT AAT GTA AGC ACC ACA ACC ACT GGT TTT CCT CTT GCC TGT GTA GGC TGG AGC TGC TTC
	ipaB rev	TCA AGC AGT AGT TTG TTG CAA AAT TGC TTT TGC AAC ATC AGT CAT ATG AAT ATC CTC CTT AG
Confirmation	ipaB-k-c-fwd	CGG TTG AAA GCC CCC TTA AAA GCT
	ipaB-k-c-rev	TGC GCT GCA ATC TGC TGA TAA TTT
**pBAD18 cloning**		**Sequence (5′ → 3′)**
pBAD fwd		ATG CCA TAG CAT TTT TAT CC
pBAD rev		CGT TCT GAT TTA ATC TGT ATC AGG
pBAD-IpaD-HA		Sequence (5′ → 3′)
pBAD-IpaDHA F EcoRI		GGG GGA ATT CTG TAA GGA AAT AAC CAT GAA TAT AAC AAC TCT G
pBAD-IpaDHA R SphI		GGG GCA TGC TCA AGC GTA GTC TGG GAC GTC GTA TGG
pBAD-IpaD-HA Q148A		Sequence (5′ → 3′)
pBAD-IpaD-HA Q148A F		GCA TAT CTG AAA GTA TAT GAA CAT GCC GTT AG
pBAD-IpaD-HA Q148A R		CTA ACG GCA TGT TCA TAT ACT TTC AGA TAT GCT TCA TTA ATA TCA TTG ATG GAG TTT GC
pBAD-IpaD-HA K151A		Sequence (5′ → 3′)
pBAD-IpaD-HA K151A F		GCA GTA TAT GAA CAT GCC GTT AGT TCA TAT ACT
pBAD-IpaD-HA K151A R		AGT ATA TGA ACT AAC GGC ATG TTC ATA TAC TGC CAG ATA CTG TTC ATT AAT ATC ATT GAT GGA G
pBAD-IpaD-HA H155A		Sequence (5′ → 3′)
pBAD-IpaD-HA H155A F		GCA GCC GTT AGT TCA TAT ACT CAA ATG TAT CAA G
pBAD-IpaD-HA H155A R		CTT GAT ACA TTT GAG TAT ATG AAC TAA CGG CTG CTT CAT ATA CTT TCA GAT ACT GTT CAT TAA TAT CAT TGA TGG AG
pBAD-IpaD-HA Y212A		Sequence (5′ → 3′)
pBAD-IpaD-HA Y212A F		GCA CCA GCA AAT AAT ACT GTT AGT CAG GAA
pBAD-IpaD-HA Y212A R		TTC CTG ACT AAC AGT ATT ATT TGC TGG TGC TAG CGG TTT ATC TTT ATA TTT TTC CTT GAG TTC

### Biofilm assay

Biofilm formation was quantified by crystal violet staining with modifications to the original protocol (75). Briefly, isolated *S. flexneri* colonies were resuspended in LB, and 10 µL of colony suspension was used to inoculate 1 mL of TSB supplemented with the indicated LCA or DCA concentrations, prepared as described above. A 150 µL aliquot of inoculated medium was dispensed into each assigned well of a flat-bottom, non-treated polystyrene 96-well plate (Genesee Scientific, 25-104; five wells per condition); edge wells and unassigned wells were filled with 150 µL sterile water to minimize evaporation. Plates were sealed with parafilm and incubated statically at 37°C for 24 h. Following incubation, spent medium was removed, and wells were washed twice with 200 µL PBS to remove loosely associated bacteria. Wells were stained with 150 µL of 0.5% (wt/vol) crystal violet (CV; Sigma-Aldrich, C0775) for 5 min at room temperature, after which the CV solution was removed, and wells were washed twice with 200 µL distilled water. Plates were air-dried for 1 h in a chemical fume hood, and CV was solubilized by the addition of 200 µL of 95% (vol/vol) ethanol, followed by thorough mixing. A 125 µL aliquot was transferred to a fresh 96-well plate, and absorbance was measured at OD_540_ using the microplate reader (Synergy H1, BioTek), with 95% ethanol as the blank.

### Sedimentation assay

Isolated *S. flexneri* colonies were resuspended in LB, and 40 µL of suspension was used to inoculate 4 mL of TSB supplemented with the indicated LCA or DCA concentrations in 15 mL culture tubes. Cultures were incubated statically at 37°C for 24 h. TSB alone or TSB supplemented with DMSO served as controls that exhibit bile acid-independent basal aggregation or bacterial pellet, hereafter referred to as the non-aggregating control. After incubation, a 150 µL aliquot was collected from the culture surface (planktonic-enriched fraction). The remaining culture was then vortexed to fully resuspend aggregates, and a separate 150 µL aliquot was taken as the total culture. OD_600_ values were measured for both the planktonic and total fractions using the microplate reader (Synergy H1, BioTek), and relative aggregation percentage was calculated as:


$$Aggregation\;\%=\frac{\left(OD_{total}-OD_{planktonic}\right)}{OD_{total}}\;x\;100$$

To enumerate CFUs, cultures were grown in parallel under the same conditions used for OD_600_ measurements. From each culture, 100 µL of the planktonic-enriched fraction was collected from the surface and plated on LB agar supplemented with Congo red. The remaining culture was then vortexed to fully resuspend sedimented bacteria, and 100 µL of this total fraction, containing both planktonic and dispersed aggregate bacteria, was plated on LB agar supplemented with Congo red. For the non-aggregating control, the same procedure was applied: 100 µL of the planktonic-enriched fraction and 100 µL of the vortexed total fraction were collected and plated separately. CFU-based aggregation ratios were calculated using the same approach as OD_600_-based aggregation ratios, following the formula provided. For proteinase K treatment, culture media were supplemented with 100 µg/mL proteinase K (QIAGEN, 19131) prior to bacterial inoculation.

### Congo red-induced effector secretion and Western blot detection

IpaD-HA secretion by the T3SS was induced with Congo red as described previously (76). Where indicated, arabinose was added at a final concentration of 0.1% (wt/vol) at the start of culture to induce IpaD-HA expression; cultures lacking arabinose served as uninduced controls. Briefly, 2 mL of Δ*ipaD* pIpaD-HA cultures at the exponential phase of growth were pelleted (761 × *g*, 10 min) and resuspended in 500 µL sterile PBS with and without 0.05% (wt/vol) Congo red. Following incubation at 37°C for 1 h, samples were centrifuged (16,100 × *g*, 30 s) to separate supernatants from pellets. Supernatants were precipitated with 10% trichloroacetic acid (Sigma-Aldrich, T0699-100ML) on ice for 1 h. Bacterial pellets were resuspended in 500 µL PBS and mixed 1:1 with 2× Laemmli sample buffer (Bio-Rad, 1610737) containing 0.1 M dithiothreitol (Fisher Scientific, FERR0861), followed by boiling for 10 min. Proteins were resolved on 12% SDS-polyacrylamide gels, transferred to PVDF membranes (BioRad, 1620177), and blocked in 5% bovine serum albumin (BSA; Fisher BioReagents, BP1600-100) in PBS containing 0.1% Tween 20 (PBST) (Sigma-Aldrich, P7949-100ML) for 1 h at room temperature. Membranes were incubated overnight at 4°C with anti-HA monoclonal antibody (Proteintech, 66006-2-Ig; 1:10,000) in 5% BSA-PBST, washed, and probed with HRP-conjugated goat anti-mouse IgG (BioRad, 1706516; 1:50,000) for 1 h at room temperature. Protein bands were visualized using Pierce ECL Western Blotting Substrate (Fisher Scientific, PI32209) and imaged with a Bio-Rad ChemiDoc system. Fiji (ImageJ) was used for quantification.

### Confocal imaging of aggregates

Preparation and inoculation for LCA-supplemented cultures followed the sedimentation assay protocol, with cultures grown for either 6 or 24 h. For complementation, cultures of the Δ*ipaD* pIpaD-HA strain were supplemented with arabinose at 0, 0.1, 0.3, and 0.5% (wt/vol) to induce expression of plasmid-borne IpaD-HA. A 50 µL aliquot was withdrawn from the aggregate layer using a wide-bore pipette tip and transferred into 500 µL of PBS containing SYTO9 (ThermoFisher Scientific, S34854; 1:1,000). Samples were incubated for 15 min at room temperature, transferred into 8-well µ-Slides (Ibidi, 80806), and allowed to settle for 20 min at 37°C. The staining solution was then aspirated and replaced with fresh PBS containing SYTO9 to maintain fluorescence. For 24-h cultures, 3D Z-stack images of WT and Δ*ipaD* strains were acquired using a Leica STELLARIS DMI8 confocal microscope equipped with a 20× water-immersion objective, with all images acquired using the same exposure settings. 2D images of 24-h Δ*ipaD* pIpaD-HA aggregates were also acquired with a 20× water-immersion objective, covering approximately 35% of the well surface randomly. Quantification was performed using the Integrated Morphometry Analysis module in ImageXpress software (Molecular Devices), with thresholds uniformly applied across all images to remove background signal and noise. To quantify 6-h aggregate size, 2D images of WT, Δ*ipaD*, and Δ*ipaD* pIpaD-HA aggregates were imaged with a 40× oil-immersion objective using LAS-X spiral acquisition, covering 40% of the well surface randomly. Using ImageXpress software (Molecular Devices), aggregate areas were quantified at three size thresholds: 1,000 px^2^ (~10 bacteria), 2,000 px^2^ (~20 bacteria), and 4,000 px^2^ (~40 bacteria). For each threshold, the following metrics were measured: total aggregate area, number of aggregates, and aggregate size. All imaging experiments were performed with at least three independent biological replicates.

### Aggregate adherence and invasion in epithelial cell monolayers

After 24 h of static growth in TSB supplemented with 50 µM LCA or 0.04% DMSO (control), 50 µL of the aggregate fraction or the control bacterial pellet, representing LCA-independent basal aggregation used as the non-aggregating control, was collected using a manually cut pipette tip. Bacterial samples were applied to 4-day confluent HT-29 or Caco-2 monolayers at MOI 200 in 24-well plates (Fisher Scientific, NC1713666) containing 500 µL of fresh McCoy’s 5A medium (for HT-29) or DMEM (for Caco-2), each supplemented with 10% heat-inactivated FBS. Plates were centrifuged for 2 min at 800 rpm to promote contact between bacteria and epithelial cells, then incubated at 37°C with 5% CO_2_. For adherence assays, monolayers were incubated for 15, 30, or 60 min before washing three times with PBS to remove non-adherent bacteria. For invasion assays, parallel wells were incubated for 15, 30, or 60 min to allow adherence, then the medium was removed and replaced with fresh media containing 50 µg/mL gentamicin (Sigma-Aldrich, NC0363642). Plates were incubated at 37°C with 5% CO_2_ for 1 h. After incubation, monolayers were washed three times with DPBS and lysed with ice-cold 0.1% Triton X-100 (Bio-Rad, 1610407) for 12 min to release intracellular bacteria. Lysates were serially diluted and plated on LB agar plates supplemented with Congo red to enumerate CFUs. Percent adhesion was calculated as the number of CFUs recovered after each infection time point (adherence step) divided by the total CFUs in the corresponding inoculum (aggregate or non-aggregating control). Percent invasion was calculated as the number of CFUs recovered after 1-h incubation with gentamicin at each time point divided by the total CFUs in the corresponding inoculum.

### Confocal imaging of aggregate-cell interactions

*S. flexneri* strains harboring the pTSAR 3.4t plasmid (52), which constitutively express mCherry and produce EGFP upon T3SS activation, were grown for 24 h under static conditions in TSB supplemented with 50 µM LCA or 0.04% DMSO (control). The LCA-induced aggregate fraction, or the bacterial pellet from the non-aggregating control, was collected with a wide-bore pipette tip and applied to 4-day confluent HT-29 and Caco-2 monolayers grown on 1.5 glass coverslips (Fisher Scientific, NC1418755) at different MOIs (20 and 200). Plates were centrifuged for 2 min at 800 rpm to facilitate contact between bacteria and epithelial cells, then incubated at 37°C with 5% CO_2_ for 15 or 30 min. After incubation, coverslips were removed, immediately fixed with 4% paraformaldehyde (Fisher Scientific, 50-980-487) for 15 min, and washed three times with DPBS. Fixed samples were incubated with Alexa Fluor-680 Phalloidin (Thermo Fisher Scientific, A22286; 1:1,000) to visualize F-actin and assess ruffling at bacteria-cell contact sites. Coverslips were mounted with ProLong Glass Antifade Mountant (Thermo Fisher Scientific, P36980), and images were acquired using a Leica STELLARIS DMI8 confocal microscope equipped with a 63× oil-immersion objective. In each experiment, 20–25 infection foci were randomly selected across each coverslip to avoid sampling bias. Image analysis was performed using LAS-X software (Leica) to quantify: (i) the area of individual F-actin foci at aggregate-cell or non-aggregating control-cell interfaces, (ii) the total mCherry fluorescence associated with each focus (representing either LCA-induced aggregates or the basal, LCA-independent bacterial pellet used as the non-aggregating control), and (iii) the corresponding total EGFP fluorescence intensity to assess T3SS activation. Normalized T3SS activity was calculated as EGFP/mCherry for each focus, thereby correcting for bacterial signal and enabling direct comparison of T3SS activity across aggregate and non-aggregating control conditions.

### *In silico* docking

The IpaD protein from *S. flexneri* was modeled using AlphaFold2 implemented via the ColabFold platform (77, 78). ColabFold was run on the DEAC high-performance computing cluster using default parameters, with no custom templates or distance restraints provided. Five models were generated, and the highest-confidence prediction (ranked by predicted LDDT) was selected as the receptor structure for docking. The selected AlphaFold2 model was prepared and converted to PDBQT format using AutoDock Tools (ADT) v.1.5.4 (79). The 3D structure of LCA (PubChem Compound CID: 9903) was retrieved from the PubChem database in SDF format (80). Ligand preparation was performed with ADT; all hydrogen atoms were added, and Gasteiger partial charges were assigned to the LCA molecule, and non-polar hydrogens were merged into a PDBQT file. Molecular docking of LCA to IpaD was carried out using AutoDock Vina (v. 1.5.2) (81). The docking search space was defined as a grid box of 50 Å per side, centered on the known DCA binding site of IpaD. All docking runs used the default Vina scoring function and search parameters and were performed on the DEAC cluster. The top nine binding poses were generated by Vina, and the highest-ranked pose (lowest predicted binding free energy) was selected for further analysis. AutoDock Vina was run in default exhaustiveness mode, and results were analyzed using VMD (v.1.9.4) (82).

### Statistical analysis

All experiments were performed with at least three independent biological replicates. Statistical analyses were conducted using GraphPad Prism 10 (GraphPad Software). Two-way ANOVA was applied for Fig. 1A, B, C, 2B, 4C, and 6; Fig. S1, S2, S3, S4, S5, S6, and S10. Three-way ANOVA with Tukey’s post hoc test was used for Fig. 3B and C. One-way ANOVA with Tukey’s post hoc test was used for aggregate quantifications in Fig. 1A and 5 and Fig. S8 and S9. Nested t-tests were used for aggregate area quantifications in Fig. S7C to account for the hierarchical structure of individual aggregate measurements nested within biological replicates. A paired t-test was applied to replicate-level means in the same figure to account for within-experiment pairing. For all figures, statistical significance thresholds are as follows: *, *P* < 0.05; **, *P* < 0.01; ***, *P* < 0.001; ****, *P* < 0.0001; ns, not significant, *P* > 0.05. Raw data and statistical analyses are provided in Supplementary Data File 1.

## SUPPLEMENTAL MATERIAL

Supplemental figures iai.00665-25-s0001.pdf Fig. S1 to S11.

Supplemental data iai.00665-25-s0002.xlsx Data sets and statistical analyses.

ASM does not own the copyrights to Supplemental Material that may be linked to, or accessed through, an article. The authors have granted ASM a non-exclusive, world-wide license to publish the Supplemental Material files. Please contact the corresponding author directly for reuse.
