# Supplementary material for: Lithocholic acid induces T3SS-dependent formation of invasion-competent Shigella flexneri aggregates
Source: Infect Immun. 2026 May 29;94(7):e00665-25. doi: 10.1128/iai.00665-25 (PMC13367064; doi:10.1128/iai.00665-25)
Supplement: Supplemental figures — Fig. S1 to S11. [file iai.00665-25-s0001.pdf]

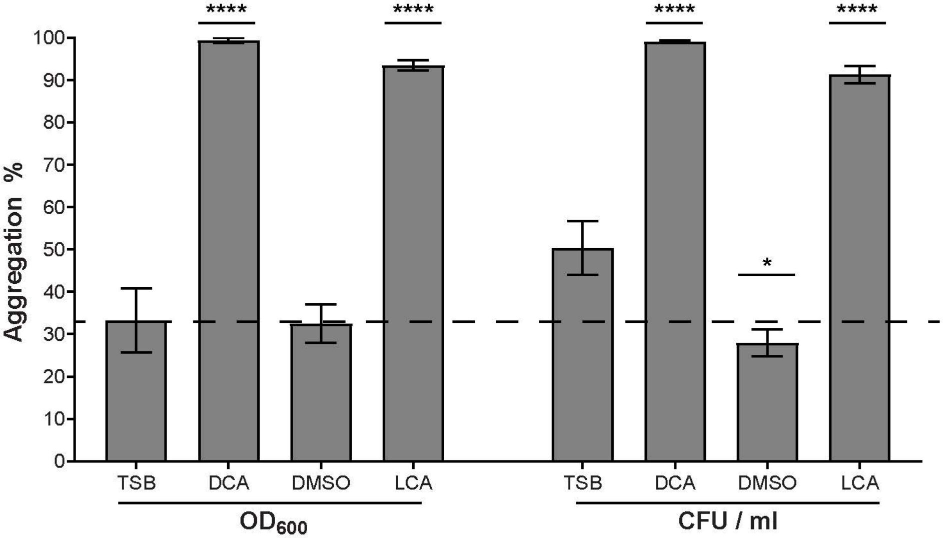

Figure S1. Comparative quantification of aggregation by OD_{600} and CFUs following the sedimentation assay. Sedimentation assay of *S. flexneri* in TSB containing DCA (2500 µM), LCA (50 µM), and vehicle for LCA (DMSO). Graph shows mean ± SD from three biological repeats. Statistics: Two-way ANOVA with Tukey's multiple comparison test, TSB as the control for each quantification. *, $p < 0.05$; ****, $p < 0.0001$.

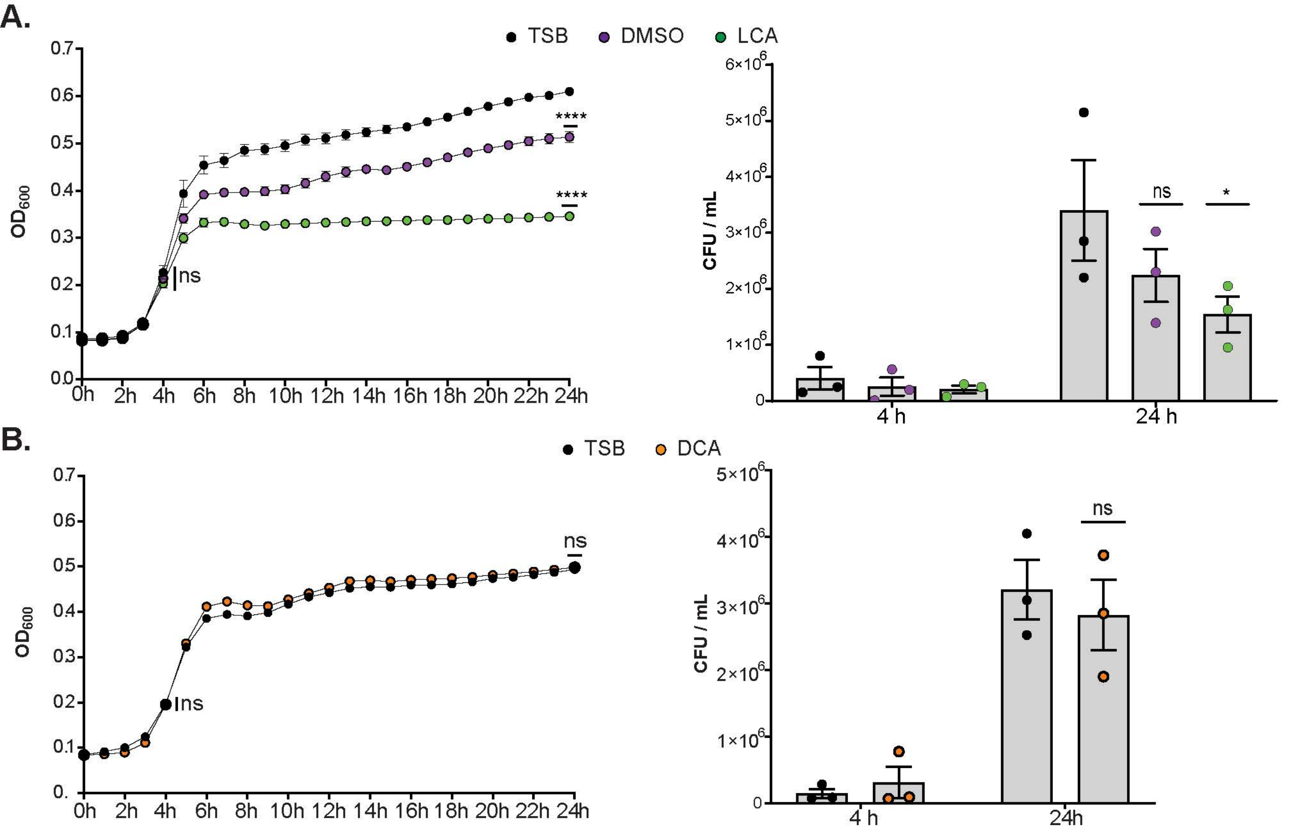

Figure S2. LCA has no impact on exponential growth while causing a subtle reduction in bacterial load at stationary phase of *S. flexneri*. Left: Growth curves of static *S. flexneri* cultures grown ± 50 µM LCA (A) or ± 50 µM DCA (B) at 37°C; OD_{600} was measured hourly for 24 h. Right: CFUs were enumerated at 4 h and 24 h to validate OD_{600} measurements. Graphs show mean ± SEM from three biological repeats. Statistics: Two-way ANOVA with (A) Tukey's multiple comparison, (B) Šídák's multiple comparison, test relative to the control at each time point. *, $p < 0.05$; ****, $p < 0.0001$; ns, not significant ($p > 0.05$).

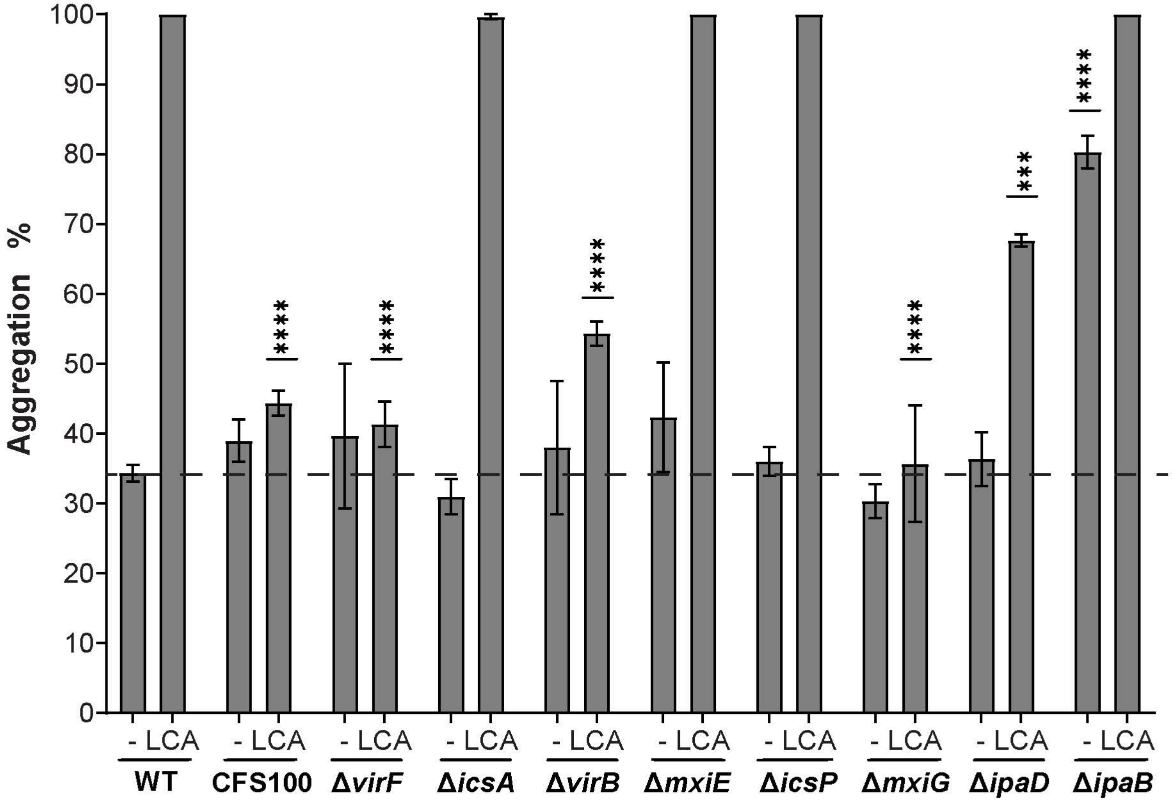

Figure S3. LCA at 100 μM induces *S. flexneri* aggregation via the T3SS and IpaD. Sedimentation assay of *S. flexneri* mutant strains grown in TSB supplemented with 100 μM LCA or DMSO vehicle (−). Graph shows mean ± SD from three biological repeats. Dashed line indicates basal bile acid-independent aggregation. Statistics: Two-way ANOVA with Tukey's multiple comparison test relative to the corresponding WT control (WT/- vs Mutant/- and WT/LCA vs Mutant/LCA). ***, $p < 0.001$; ****, $p < 0.0001$.

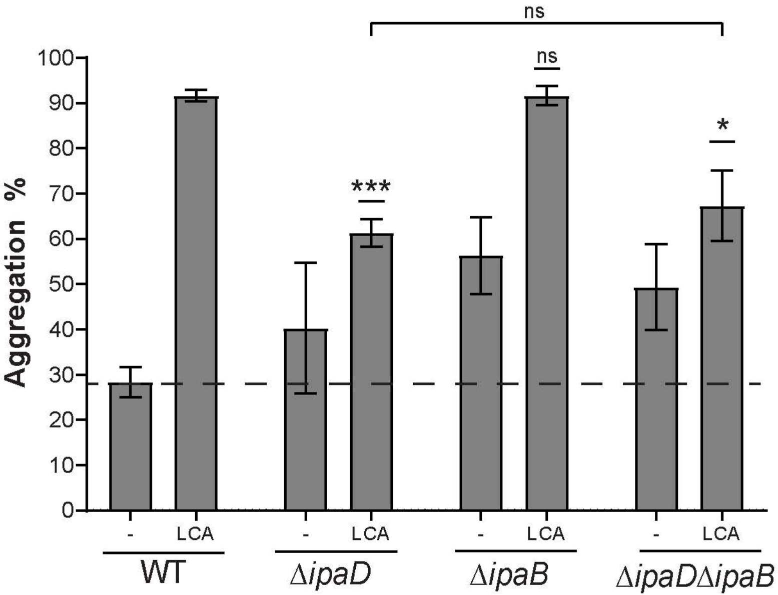

Figure S4. The Δ*ipaD* Δ*ipaB* mutant is defective in LCA-induced aggregation. Sedimentation assay of WT, Δ*ipaD*, Δ*ipaB*, and Δ*ipaD* Δ*ipaB* strains grown in TSB supplemented with 50 µM LCA or DMSO vehicle (−). Graph shows mean ± SD from three biological repeats. Dashed line indicates basal bile acid-independent aggregation. Statistics: Two-way ANOVA with Tukey's multiple comparison test relative to the corresponding WT control while bracket shows comparision between Δ*ipaD* and Δ*ipaD*Δ*ipaB* . *, $p < 0.05$; ***, $p < 0.001$; ns, not significant ($p > 0.05$).

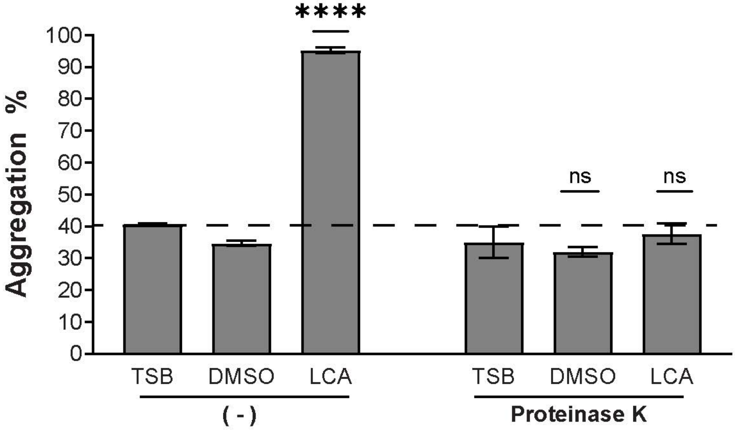

Figure S5. Proteinase K sensitivity of LCA-induced aggregation. Sedimentation assay performed in TSB and TSB supplemented with DMSO or 50 μM LCA ± proteinase K. Graph shows mean ± SD from three biological repeats. Dashed line indicates basal bile acid-independent aggregation. Statistics: Two-way ANOVA with Tukey's multiple comparison test relative to DMSO control. ****, $p < 0.0001$; ns, not significant ($p > 0.05$).

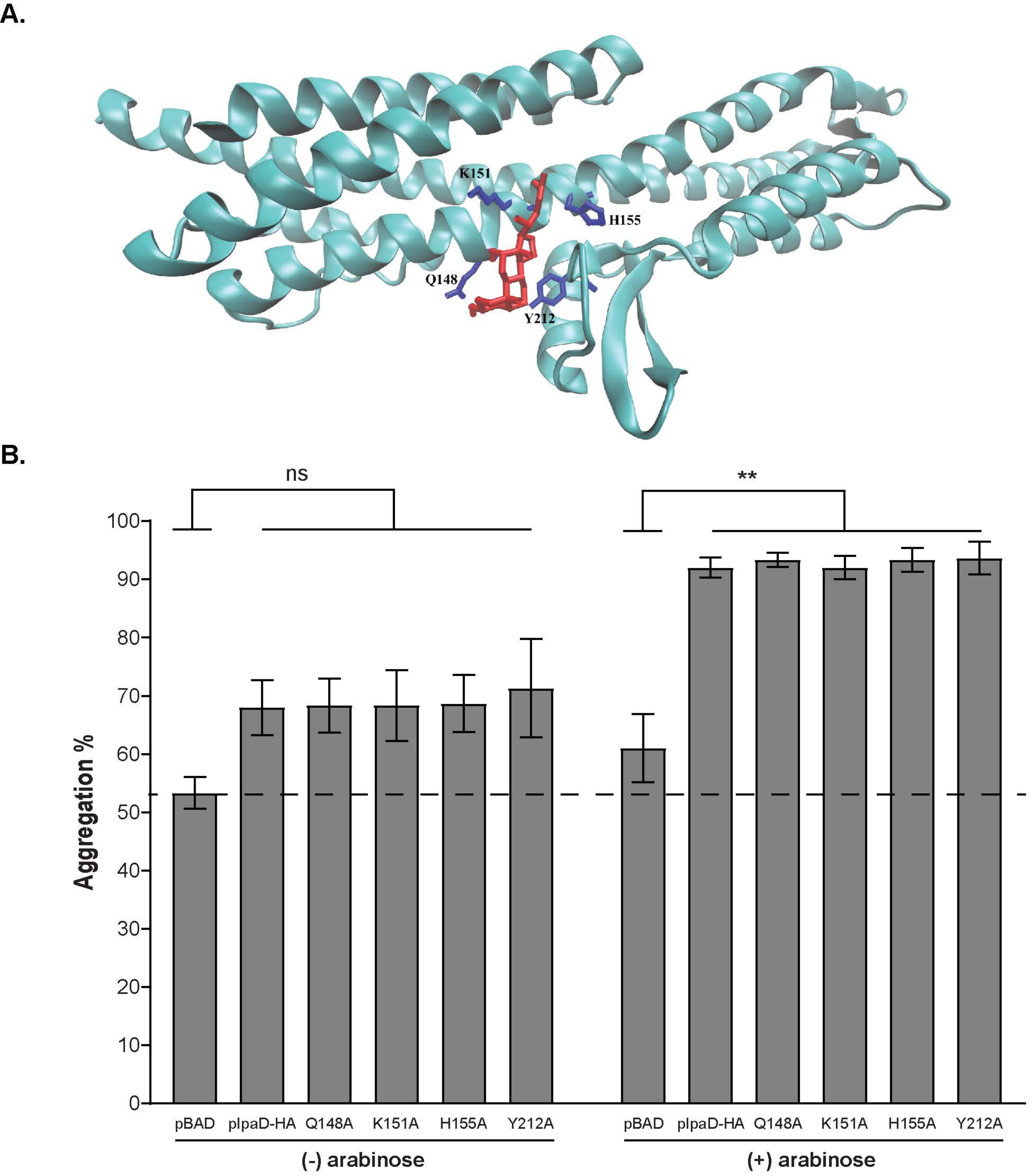

Figure S6. Potential IpaD residues interacting with LCA. (A) AlphaFold-predicted structure of IpaD rendered in NewCartoon representation (cyan), with LCA (docked via AutoDock Vina) shown in red bonded representation and predicted ligand-interacting residues Q148, K151, H155, and Y212 highlighted in blue bonded representation. Visualization generated with VMD 1.9.4. (B) Sedimentation assay with Δ*ipaD* mutant harboring pBAD18 (Ctrl), pIpaD-HA (WT), and single alanine substitutions at Q148, K151, H155, and Y212 residues ± 0.2% arabinose. Graph shows mean ± SD from three biological repeats. Statistics: Two-way ANOVA with Tukey's multiple comparison test. **, $p < 0.01$; ns, not significant ($p > 0.05$).

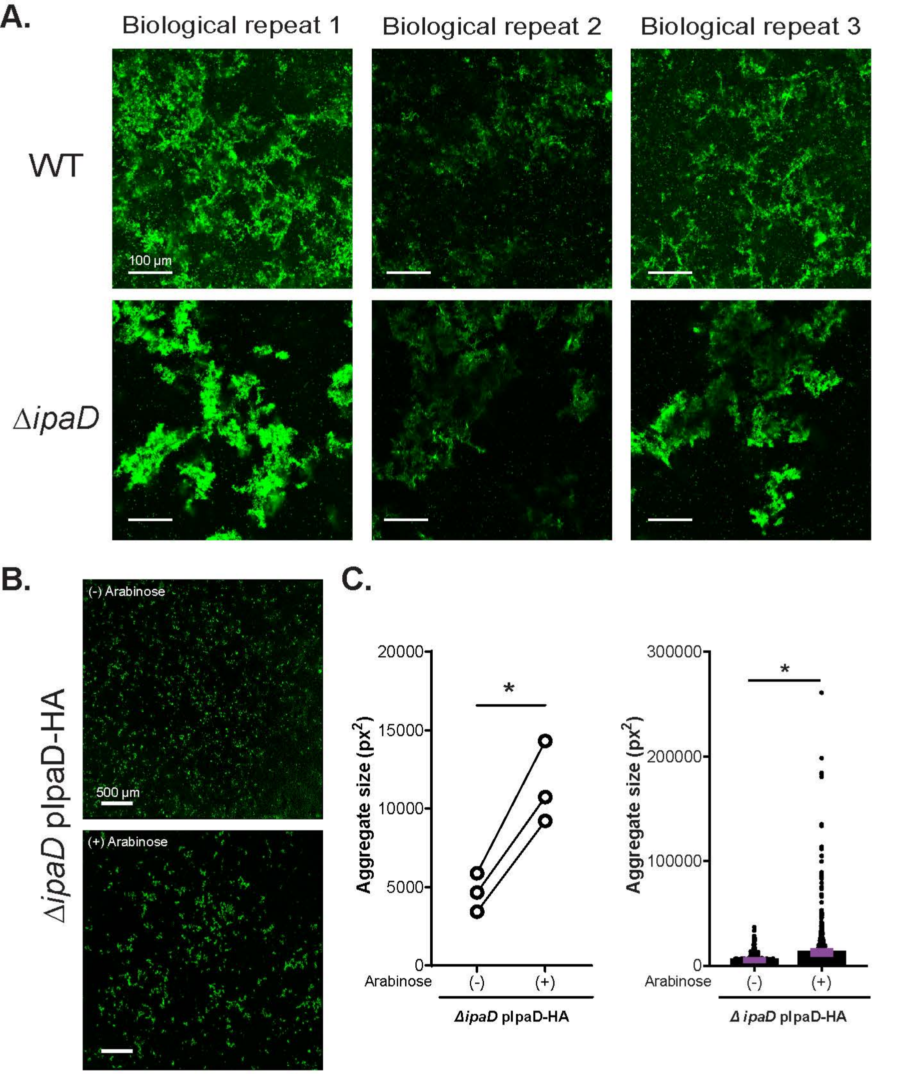

Figure S7. **IpaD influences aggregate size in LCA-induced aggregation at 24 h.** (A) Widefield images of WT and Δ*ipaD* sedimented aggregates after 24 h static growth with 50 µM LCA, stained with SYTO-9 from three biological repeats. Images were acquired with 20x objective. (B) Representative widefield images (20x objective) of Δ*ipaD* pIpaD-HA sedimented aggregates after 24 h growth with 50 µM LCA in the absence (−) and presence (+) of 0.1% (w/v) arabinose, stained with SYTO-9. Scale bar, 500 µm. (C) Quantification of aggregate sizes. Left: Graph shows replicate-level means of projected aggregate area from three biological repeats; each circle represents one biological repeat, and lines connect paired samples within the same experiment. Statistics: paired t-test applied to replicate-level means to account for within-experiment pairing. Right: Graph shows the distribution of all individual aggregate areas measured across three biological repeats. Statistics: nested t-test applied to account for the hierarchical structure of individual aggregate measurements nested within biological replicates. *, $p < 0.05$.

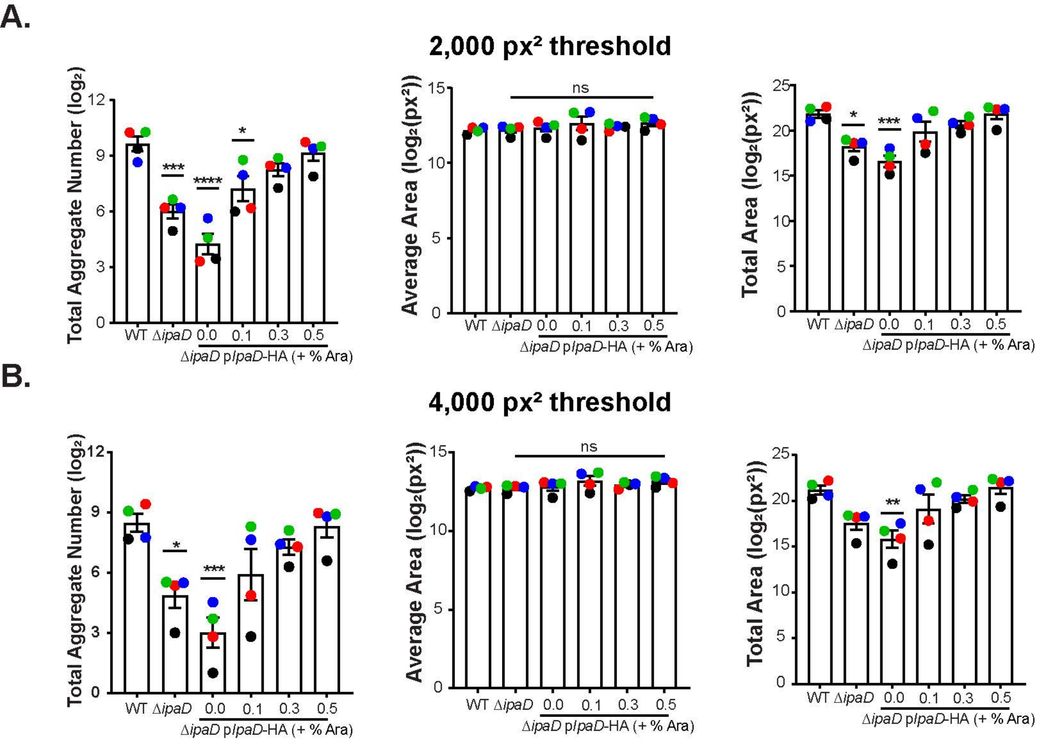

Figure S8. Aggregate quantifications at 2,000 and 4,000 px² minimum aggregate area thresholds. Quantification of aggregate number, total aggregate area, and aggregate size for WT, Δ*ipaD*, and Δ*ipaD* pIpaD-HA grown with increasing arabinose levels after 6 h with 50 µM LCA, using minimum area thresholds of (A) 2,000 px² and (B) 4,000 px². Graphs show mean ± SD from four biological repeats indicated by different colors. Statistics: One-way ANOVA with Tukey's multiple comparison test, WT as the control. *, $p < 0.05$; **, $p < 0.01$; ***, $p < 0.001$; ****, $p < 0.0001$; ns, not significant ($p > 0.05$).

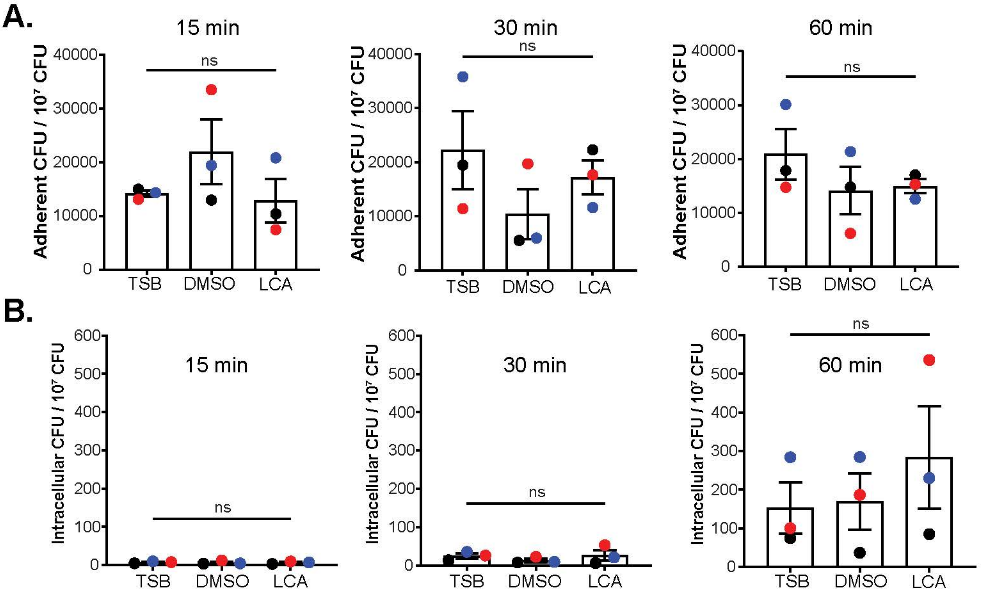

Figure S9. LCA-induced bacterial aggregates adhere to and invade Caco-2 cells. (A) Adhesion to Caco-2 cells at MOI 200 by non-aggregating controls grown in TSB, TSB + vehicle (DMSO), and LCA-induced aggregates (LCA) at 15, 30, and 60 min post-infection. (B) Invasion of Caco-2 cells at MOI 200 by non-aggregating controls grown in TSB, TSB + vehicle (DMSO), and LCA-induced aggregates (LCA) at 15, 30, and 60 min post-infection. Graphs show mean ± SD from three biological repeats indicated by different colors. Statistics: One-way ANOVA with Tukey's multiple comparison test. ns, not significant ($p > 0.05$).

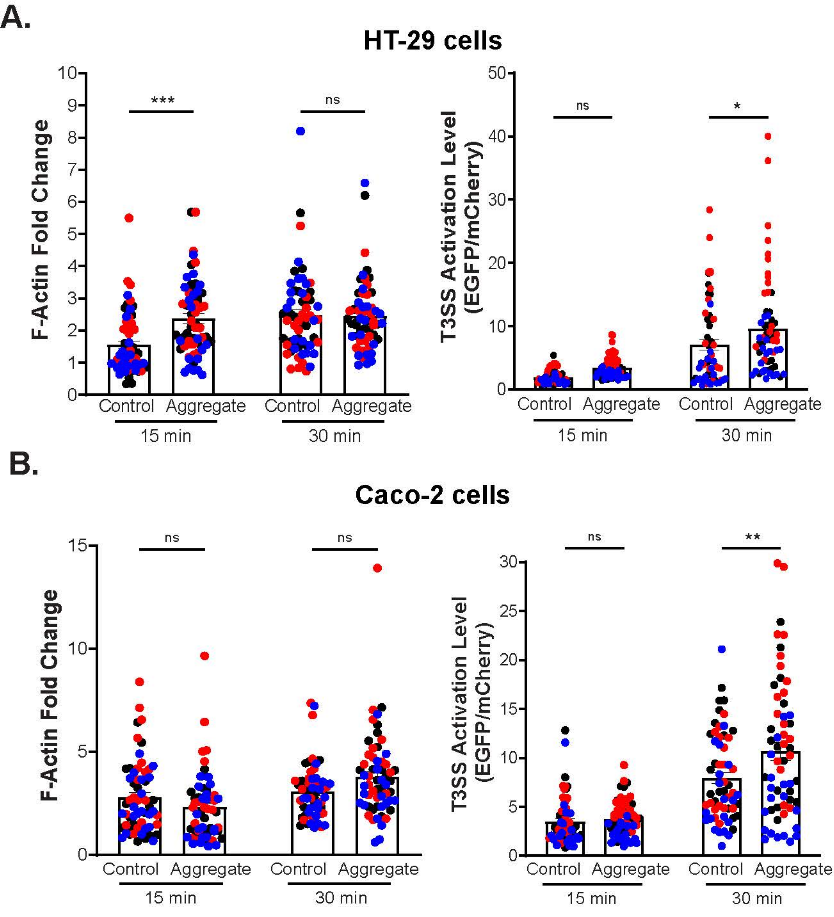

Figure S10. LCA-induced aggregates confer comparable actin polymerization and increased T3SS activity at MOI 200 . (A) HT-29 and (B) Caco-2 cells, infected with non-aggregating controls (Control) or LCA-induced aggregates (Aggregate) at 15 and 30 min post-infection. Left Graph: Quantification of F-actin focus intensity via normalization to uninfected cell regions. Right Graph: Quantification of EGFP fluorescence intensity via normalization to mCherry fluorescence intensity per infection focus, reporting T3SS activity per bacterial signal. Graphs show mean ± SEM from three biological repeats indicated by different colors. Statistics: Two-way ANOVA with Tukey's multiple comparison test. *, $p < 0.05$; **, $p < 0.01$; ***, $p < 0.001$; ns, not significant ($p > 0.05$).

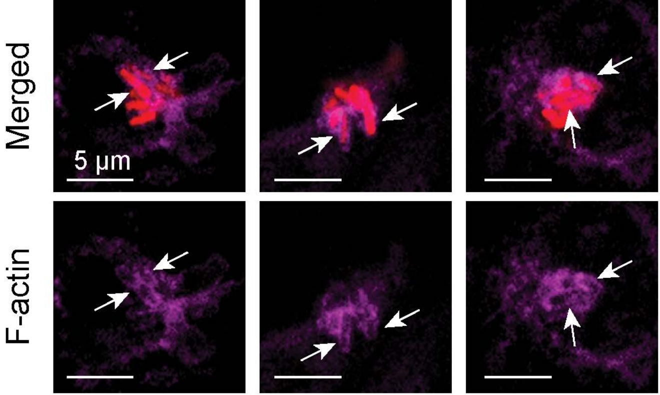

Figure S11. Infection with LCA-induced aggregates results in invasion vacuole formation within HT-29 cells. Representative images of 30 min infections at MOI 20 with *S. flexneri* grown in TSB containing 50 µM LCA in HT-29 monolayers. Merged image, representative single Z-plane; *S. flexneri* (mCherry) and F-actin (magenta). White arrows indicate vacuole-associated bacteria. Scale bar, 5 µm.
